# ﻿Pathogenic fungi (Sordariomycetes) associated with annual and perennial crops in Northern Thailand

**DOI:** 10.3897/mycokeys.117.137112

**Published:** 2025-05-09

**Authors:** Maryam Fallahi, Alireza Armand, Fatimah Al-Otibi, Kevin D. Hyde, Ruvishika S. Jayawardena

**Affiliations:** 1 Center of Excellence of Fungal Research, Mae Fah Luang University, Chiang Rai, 57100, Thailand Mae Fah Luang University Chiang Rai Thailand; 2 Department of Entomology and Plant Pathology, University of Arkansas, Fayetteville, AR 72703, USA University of Arkansas Fayetteville United States of America; 3 School of Science, Mae Fah Luang University, Chiang Rai, 57100, Thailand King Saud University Riyadh Saudi Arabia; 4 Department of Botany and Microbiology, College of Science, King Saud University, P.O. Box 22452, 11495 Riyadh, Saudi Arabia5 Kyung Hee University, 26 Kyungheedae-ro, Dongdaemun-gu, Seoul, 02447, Republic of Korea Kyung Hee University Seoul Republic of Korea

**Keywords:** Diversity, new species, pathogenicity, phytopathogens, phylogenetic analysis

## Abstract

Phytopathogenic fungi induce severe diseases in plant hosts, potentially leading to significant crop quantity and quality reductions. In this study, we isolated and identified pathogenic fungi that threaten the successful cultivation of annual and perennial crops in northern Thailand. Samples with leaf spots, fruit rot, wilting, and dieback symptoms were collected from 19 different crops in Chiang Rai, Chiang Mai, and Sakon Nakhon provinces in Thailand. Out of 183 isolates, 27 were selected for molecular analysis and multi-gene phylogenetic study based on their diverse host range, geographical distribution, and an initial morphological assessment. Four of the most species-diverse fungal genera – *Colletotrichum*, *Diaporthe*, *Fusarium*, and *Neopestalotiopsis* – were identified, along with 23 taxa, including four novel species. Six species of *Colletotrichum* (*Colletotrichumabelmoschi***sp. nov.**, *C.fructicola*, *C.makassarense*, *C.plurivorum*, *C.siamense*, and *C.spaethianum*), six species of *Diaporthe* (*Diaporthefistulosi***sp. nov.**, *D.hongkongensis*, *D.melongenicola***sp. nov.**, *D.rosae*, *D.sennicola*, and *D.siamensis*), five species of *Fusarium* (*Fusariumbubalinum*, *F.languescens*, *F.nirenbergiae*, *F.sulawesiense*, and *F.tanahbumbuense*), and three species of *Neopestalotiopsis* (*Neopestalotiopsisformicidarum*, *N.theobromicola***sp. nov.**, and *N.zakeelii*, along with three unspecified taxa), were isolated and characterized in this study. Additionally, fourteen new host records and eight new geographical records are reported. Pathogenicity tests were carried out for selected strains, and the results confirmed their pathogenicity on their host plants. This study offers new insights into the diversity of species of plant pathogenic fungi in northern Thailand.

## ﻿Introduction

Fungi play a crucial role in every ecosystem (Buckley 2020). They are one of the important biotic elements responsible for severe diseases in a broad range of plant hosts (Hariharan and Prasannath 2021). They lead to various diseases such as anthracnose, blight, canker, damping off, dieback, gall, leaf spot, powdery mildew, root rot, scab, and wilt in economically important crops (Marin-Felix et al. 2017; Jayawardena et al. 2019; Hariharan and Prasannath 2021; Gomdola et al. 2022; Venbrux et al. 2023).

*Colletotrichum* (Jayawardena et al. 2021; Armand et al. 2022, 2023), *Diaporthe* (Gomes et al. 2013), *Fusarium* (Gerlach and Nirenberg 1982), *Pestalotiopsis* (Hyde et al. 2014), and *Neopestalotiopsis* (Maharachchikumbura et al. 2014) are globally recognized as the most destructive plant pathogens. Additionally, toxigenic plant pathogens, such as *Fusarium*, not only cause diseases in a diverse range of hosts but can also indirectly impact human and animal health by producing mycotoxins (Moretti et al. 2017; Fallahi et al. 2019; Munkvold et al. 2021).

Accurate identification and detection of plant pathogens are crucial for effective disease management (Hariharan and Prasannath 2021; Venbrux et al. 2023). Pathogens may require diverse control strategies, such as fungicides, resistant plant varieties, or crop rotation (Juroszek and Von Tiedemann 2011; Peng et al. 2014). Regularly identifying and monitoring fungal plant pathogens helps to track their distribution and virulence changes. This information can be used for long-term disease management strategies (Juroszek and Von Tiedemann 2011).

Thailand, a tropical biodiversity hotspot (Myers et al. 2000), serves as a key agricultural hub, particularly in the north, where diverse crops are cultivated. Major crops include rice, maize, vegetables, and tropical fruits such as durian (Teh et al. 2017), mangosteen (Ongkunaruk and Piyakarn 2011), rambutan (Salakpetch 2005), dragon fruit (Kakade et al. 2022), and guava (Suwanwong and Boonpangrak 2021). The region also produces coffee, tea (Vichitrananda and Somsri 2008), cacao (Chaisu and Chiu 2019), and black pepper (Ravindran and Kallupurackal 2012; Mokshapathy 2013). Multipurpose crops such as jackfruit (Mandal et al. 2023; Rahim 2023) and tamarind (Kumar and Bhattacharya 2008; Noraphaiphipaksa et al. 2016) support food, timber, and pharmaceutical industries. Other significant crops include edible canna (Piyachomkwan et al. 2002), persimmons (Satheinkhot et al. 2004), sapodilla (Poonsawat et al. 2007), lemon drop mangosteen (Acuña 2011), okra (Benchasri and Benchasri 2012), *Allium* species (Saensouk and Saensouk 2021), makhuea kheun (Hussain et al. 2017; Kumar et al. 2020), lesser yam (Rugchati and Thanacharoenchanaphas 2010), and pepper (Laprom et al. 2019). However, the thriving agricultural sector in Thailand faces various challenges, notably the presence of pathogenic fungi that can significantly impact crop health and yield (Kodsueb et al. 2021; Armand et al. 2023). Studies on fungi associated with tropical hosts are limited, and this is the main reason for poor knowledge of fungal diversity in tropical areas (Hyde et al. 2018). Previous studies of fungi in northern Thailand have proposed a novelty of fungi between 55% and 96% (Hyde et al. 2018). This study was conducted as a continuation to discover fungi associated with tropical and subtropical crops in northern Thailand.

## ﻿Materials and methods

### ﻿Sample collection, examination, and isolation

From 2022 to 2023, samples with leaf spots, rotting fruits, wilting, and dieback of 13 diverse perennial crops, including black pepper (*Pipernigrum* L.), Brazil cherry (*Eugeniabrasiliensis* Lam.), cacao (*Theobromacacao* L.), dragon fruit (*Hylocereustrigonus* (Haw.) Sarg.), durian (*Duriozibethinus* L.), guava (*Psidiumguajava* L.), jackfruit (*Artocarpusheterophyllus* Lam.), lemon drop mangosteen (*Garciniaintermedia* (Pittier) Hammel), mangosteen (*Garciniamangostana* L.), persimmon (*Diospyrosehretioides* Wall.), rambutan (*Nepheliumlappaceum* L.), sapodilla sapote (*Manilkarazapota* (L.) P. Royen), and tamarind (*Tamarindusindica* L.) were collected from orchards in Chiang Rai, Chiang Mai, and Sakon Nakhon provinces in Thailand (Suppl. material 1).

Additionally, six annual crops, including edible canna (*Cannaindica* L.), lesser yam (*Dioscoreaesculenta* (Lour.) Burkill), makhuea (*Solanummelongena* L.), okra (*Abelmoschusesculentus* (L.) Moench), pepper (*Capsicumannuum* L.), and spring onion (*Alliumfistulosum* L.), were also collected from farms in the same regions (Suppl. material 1).

Specimens were transferred to the laboratory in Ziploc plastic bags for examination. Specimen fragments, each measuring 3 mm^2^, were carefully removed from the periphery of the lesions to obtain tissue samples. Subsequently, they were sterilized by immersing them in 70% ethanol for 2 minutes, followed by a 10% sodium hypochlorite solution for 60 seconds. They were then rinsed thrice with sterile distilled water, each lasting 1 minute. Subsequently, they were transferred to a petri dish with potato dextrose agar medium (PDA), and after 4–5 days of post-incubation, the fungi grown from the tissue segment edges were subcultured to a fresh PDA. The single-spore and hyphal tip isolation methods were employed for fungal isolation and purification (Senanayake et al. 2020). Pure cultures were deposited in the Mae Fah Luang University Culture Collection (MFLUCC) in Chiang Rai, Thailand. The dried cultures were stored in the Mae Fah Luang University Herbarium (MFLU).

### ﻿Morphological identification

For morphological characterization, pure cultures were transferred onto potato dextrose agar (PDA), carnation leaf-piece agar (CLA, 20.0 g/L agar prepared in distilled water and a piece of the disinfected carnation leaf), and synthetic nutrient agar (SNA, 1.0 g/L of KH2PO4, 1.0 g/L of KNO3, 0.5 g/L of MgSO4·7H2O, 0.5 g/L of KCl, 0.2 g/L of glucose, 0.2 g/L of sucrose, and 20.0 g/L agar). The fruiting bodies and fungal structures that developed on the PDA, SNA, and CLA media were observed and photographed using a stereo-microscope (OLYMPUS-SZX16). Morphological characters were observed using the LEICA-EZ4 stereo-microscope and photographed with an optical microscope with a Nikon DS-Ri2 camera. All measurements were carried out using image framework Version 0.9.7, and images were processed with Adobe Photoshop CS6 v. 13.1.2 (Adobe Systems, USA).

### ﻿DNA extraction, PCR amplification, and sequencing

Genomic DNA was extracted from fresh mycelia cultured on PDA for 10 days using the DNA Extraction Kit (Omega Bio-Tek, USA), following the manufacturer’s instructions. The polymerase chain reaction (PCR) was carried out in a total volume of 25 μL, comprising 12.5 μL of 2 × Power Taq PCR Master Mix, 1 μL of each primer (20 μM), 1 μL of genomic DNA, and 9.5 μL of deionized water. The information related to the forward and reverse primers and PCR conditions employed in this study is listed in Table 1. The PCR amplification was done in an Eppendorf (Master cycler X50s, Germany) thermal cycler. SolGent Co., Republic of Korea, performed the sequencing of PCR products.

**Table 1.** PCR conditions and primers that were used in this study.

Locus (Primers)	PCR conditions	Fungal genus	References
**ITS (ITS4/ITS5)**	95 °C: 5 min, (95 °C: 45 s, 53 °C: 45 s, 72 °C: 2 min) × 40 cycles, 72 °C: 10 min	all genera	(White et al. 1990)
***Actin* (ACT-512F/ACT-783R)**	95 °C: 5 min, (95 °C: 30 s, 55 °C: 50 s, 72 °C: 1 min) × 40 cycles, 72 °C: 10 min	*Colletotrichum*	(Carbone and Kohn 1999)
***Chitin synthase* (CHS79F/CHS345 R)**	94 °C: 5 min, (94 °C: 50 s, 58 °C: 30 s, 72 °C: 1:30 min) × 35 cycles, 72 °C: 10 min	*Colletotrichum*	(Carbone and Kohn 1999)
***GAPDH* (GDF/GDR)**	95 °C: 5 min, (95 °C: 50 s, 58 °C: 50 s, 72 °C: 1 min) × 40 cycles, 72 °C: 10 min	*Colletotrichum*	(Templeton et al. 1992)
***tub2* (Btub2Fd/Btub4Rd)**	94 °C: 3 min, (94 °C: 30 s, 58 °C: 1:30 min, 72 °C: 1:20 min) × 40 cycles, 72 °C: 10 min	*Colletotrichum*, *Diaporthe*	(Woudenberg et al. 2009)
***Calmodulin* (CL1/CL2A)**	95 °C: 5 min, (95 °C: 30 s, 54 °C: 50 s, 72 °C: 1 min) × 40 cycles, 72 °C: 10 min	*Diaporthe*	(O’Donnell et al. 2000)
***his3* (CYLH3F/H3-1b)**	94 °C: 3 min, (95 °C: 30 s, 57 °C: 30 s, 72 °C: 1 min) × 40 cycles, 72 °C: 10 min	*Diaporthe*	(Glass and Donaldson 1995; Crous et al. 2004)
***tef1* (EF1/EF2)**	94 °C: 3 min, (94 °C: 30 s, 58 °C: 1:30 min, 72 °C: 1:20 min) × 40 cycles, 72 °C: 10 min	*Fusarium*	(O’Donnell et al. 1998)
***RPB1* (Fa/R8)**	94 °C: 5 min, (94 °C: 30 s, 53 °C: 50 s, 72 °C: 1 min) × 35 cycles, 72 °C: 10 min	*Fusarium*	(Hofstetter et al. 2007; O’Donnell et al. 2010)
***RPB2* (5f2/7cr)**	94 °C: 3 min, (94 °C: 30 s, 58 °C: 1 min, 72 °C: 1:20 min) × 40 cycles, 72 °C: 10 min	*Fusarium*	(Reeb et al. 2004)
***tef1* (EF-728F/EF2)**	94 °C: 3 min, (94 °C: 30 s, 58 °C: 1:30 min, 72 °C: 1:20 min) × 40 cycles, 72 °C: 10 min	*Diaporthe*, *Neopestalotiopsis*	(O’Donnell et al. 1998; Carbone and Kohn 1999)
***tub2* (Bt2a/Bt2b)**	94 °C: 3 min, (94 °C: 30 s, 58 °C: 1:30 min, 72 °C: 1:20 min) × 40 cycles, 72 °C: 10 min	*Neopestalotiopsis*	(Glass and Donaldson 1995; O’Donnell and Cigelnik 1997)

### ﻿Phylogenetic analyses

The obtained sequences were subjected to a BLAST search, and matching reference sequences were acquired from GenBank, along with relevant information from recently published papers (Maryani et al. 2019a, b; Guo et al. 2020; Crous et al. 2021; Wang et al. 2021; Norphanphoun et al. 2022; Santos et al. 2022; Fiorenza et al. 2022; Alhudaib et al. 2023; Zhang et al. 2023). All the sequences were aligned using the online tool MAFFT version 7 under the web server (http://mafft.cbrc.jp/alignment/server) (Katoh et al. 2019). As required, the alignment was further adjusted using BioEdit v. 7.0.9.0 and subsequently trimmed with command-based TrimAl software automatically (Zeng et al. 2023).

Phylogenetic analyses were performed to determine the phylogenetic placement of the isolated taxa using maximum parsimony (MP), maximum likelihood (ML), and Bayesian inference on the CIPRES web portal. The nucleotide substitution model GTR+GAMMA was employed for maximum likelihood analysis using RAxMLHPC2 on XSEDE (version 8.2.12), with 1,000 bootstrap replicates. The Bayesian analysis was performed with the utilization of a Markov Chain Monte Carlo (MCMC) algorithm to estimate Bayesian posterior probabilities (BYPP) using MrBayes on XSEDE (Ronquist et al. 2012), with four independent MCMC chains from random trees for 1,000,000 generations and sampled every 100^th^ generation. The initial 25% of the generated trees were removed as burn-in, and the residual trees were used for calculating posterior probabilities.

A maximum parsimony (MP) analysis was performed using PAUP XSEDE. Gaps were considered as missing data, and areas with ambiguous alignments were excluded. The analyses were executed on the CIPRES Science Gateway (https://www.phylo.org/portal2) (Miller et al. 2011). To assess the level of recombination among the closely related species, a pairwise homoplasy index (PHI) test using Splits Tree 4 (version 4.14.2) was employed. The obtained phylograms were visualized in FigTree v. 1.4.0 (Rambaut 2014) and annotated in Adobe Illustrator CC 22.0.0 (Adobe Systems, San Jose, CA, USA).

### ﻿Species identification

Species identification was carried out following the methodologies outlined by Chethana et al. (2021), Jayawardena et al. (2021), and Maharachchikumbura et al. (2021). The process included isolation of fungal strains on appropriate culture media, performing morphological characterization using light microscopy, extracting DNA, amplifying, and sequencing key gene regions (e.g., ITS, *tef1-α*, and *tub2*). The obtained sequences were then compared with reference sequences available in GenBank and other databases to ensure accurate identification and validation of the species.

### ﻿Pathogenicity tests

Pathogenicity tests were applied to confirm the pathogenicity of the fungal isolates obtained in this study, following the methodology described by Bhunjun et al. (2021). Pathogenicity tests were conducted to evaluate the disease-causing potential of selected strains, including *Colletotrichum* (five strains), *Diaporthe* (six strains), *Fusarium* (five strains), and *Neopestalotiopsis* (five strains). *Colletotrichummakassarense* (24-0237) on leaves and fruits of tamarind, *C.abelmoschi* (MFLUCC 24-0239) on okra, *C.fructicola* (MFLUCC 24-0240) on leaves of jackfruit, *C.siamense* (MFLUCC 24-0235) on leaves of black pepper, *C.plurivorum* (MFLUCC 24-0238) on fruits of persimmon, *Diaporthesennicola* (MFLUCC 24-0241) on okra, *Diaporthefistulosi* (MFLUCC 24-0244) on spring onion, *D.hongkongensis* (MFLUCC 24-0246) and *D.siamensis* (MFLUCC 24-0245) on rambutan fruits, *D.rosae* (MFLUCC 24-0243) and *Diaporthemelongenicola* (MFLUCC 24-0242) on fruits of makhuea, *Fusariumsulawesiense* (MFLUCC 24-0250) on mangosteen fruits, *F.tanahbumbuense* (MFLUCC 24-0231) on leaves of durian and pepper fruits, *F.bubalinum* (MFLUCC 24-0230) on stem of dragon fruit, *F.nirenbergiae* (MFLUCC 24-0248) on spring onion, *Neopestalotiopsisformicidarum* (MFLUCC 24-0254) on rambutan fruits, *Neopestalotiopsis* sp. 2 (MFLUCC 24-0255) on mangosteen fruits, *Neopestalotiopsis* sp. 3 (MFLUCC 24-0251) on leaves of guava, *Neopestalotiopsiszakeelii* (MFLUCC 24-0252) on persimmon fruit, and *Neopestalotiopsis* sp. 1 (MFLUCC 24-0232) on leaves of sapodilla sapote. The pathogenicity test of *Fusariumlanguescens* on lesser yam and *Neopestalotiopsistheobromicola* on cacao was not conducted due to the unavailability of the host plants.

Artificial inoculations were performed on detached plant organs to evaluate the ability of fungi to induce disease and establish infection. Fruits and leaves that were visually healthy and without any visible discolorations, lesions, and other disease symptoms were surface sterilized to minimize the risk of contamination. The surface sterilization procedure involved immersing the plant tissues in 70% ethanol for 2 minutes, followed by a 2-minute treatment with a 2% solution of sodium hypochlorite. Afterward, the plant tissues were rinsed thoroughly with sterile distilled water and allowed to dry in a laminar flow cabinet to ensure aseptic conditions.

Both fruits and leaves were wounded to create an entry point for the fungi, as some pathogens may require physical injury to avoid the plant’s natural defense and infect the tissue. The wounding procedure was carried out using a sterile scalpel or needle to create small, consistent lesions. Small incisions of approximately 2–5 mm in diameter were made on the surface of the fruit at multiple sites (typically 3–5 wounds per fruit). For the leaves, wounds of uniform size were created at the same replications.

Fresh fungal colonies grown on PDA were used to prepare the mycelium plugs for inoculation. Control inoculations were performed using non-colonized PDA plugs. Inoculated plant tissues were then incubated individually in a moist chamber at a temperature of 28 °C with 80% humidity. The incubation period ranged from 7 to 14 days, depending on the specific pathogen and host plant.

After the incubation, the pathogen was reisolated from the lesions on the inoculated plant tissues. The identification of the fungal isolate was confirmed through morphological analysis and molecular techniques, ensuring that the pathogen responsible for the lesions was the same as the inoculated fungal isolate.

## ﻿Results

In total, 183 fungal isolates were collected in the surveyed orchards and farms across the Chiang Rai, Chiang Mai, and Sakon Nakhon provinces in Thailand, representing four prevalent genera: *Colletotrichum*, *Diaporthe*, *Fusarium*, and *Neopestalotiopsis*. Based on preliminary morphological identification, 27 isolates were selected for molecular and phylogenetic analysis and subsequently classified into 23 fungal species. Detailed information on the phylogenetic analysis, as well as descriptions and illustrations of these taxa, is provided below.

### ﻿ Colletotrichum

Taxon classificationFungiSordariomycetesGlomerellaceae

Corda, Deutschlands Flora, Abt. III. Die Pilze Deutschlands. 3 (12): 41; (1831).

8222FD11-01E9-561A-9B02-394AB291BE69

#### Notes.

This study describes five well-characterized species of *Colletotrichum*: *C.fructicola*, *C.makassarense*, *C.siamense*, *C.plurivorum*, and *C.spaethianum*, and introduces one novel species, *Colletotrichumabelmoschi*.

### ﻿ Colletotrichum abelmoschi

Taxon classificationFungiSordariomycetesGlomerellaceae

Fallahi, Jayawar. & K.D. Hyde sp. nov.

4F96B19A-E54A-5925-8576-E4AC6F1BA2EF

Index Fungorum: IF903264

Fig. 2

#### Etymology.

‘*abelmoschi*’ refers to the host plant genus where the fungus was isolated.

#### Description.

Fungus causes small, dark brown to black lesions on the okra stems. On the leaves, infection results in angular to irregular-shaped dark brown or black spots that expand into larger areas of necrosis. A yellow halo often surrounds these spots. Sexual morph not observed. Conidiomata acervular, semi-immersed, dark brown, bearing conidial masses. Conidiophore hyaline to pale brown, cylindrical or subcylindrical, branched or unbranched, septate, smooth-walled, 11–27 × 2–4.5 μm (mean = 19 × 3.3 μm, n = 30). Conidiogenous cells cylindrical or doliiform, unbranched, guttulate, 6–18 × 2.2–4 μm (mean = 11.5 × 3 μm, n = 30). Conidia aseptate, straight, cylindrical, the apex and base rounded or tapering at base, guttulate, 8.6–13.7 × 4.2–5.8 μm (mean = 12 × 5 μm, n = 40). Appressoria ellipsoidal to obovate, clavate, entire margin, regular shape, or slightly irregular, brown to dark brown 4–12 × 5–7 μm (mean = 8 × 6 μm, n = 20). Chlamydospores and Setae are absent.

#### Culture characteristics.

Colonies on PDA reach 60 mm in diameter after 7 days of growth at 25 °C in the dark, cottony, circular shape, dull surface, entire edge, well-defined margin, with medium density. Upper view white to light grey. Reverse primrose, with no diffusing pigment.

#### Material examined.

Thailand • Sakon Nakhon Province, Mueang Sakon Nakhon District, on leaf of okra (*Abelmoschusesculentus*), February 2023, Maryam Fallahi, dried culture MF148-1 (MFLU 24-0237, holotype), ex-holotype culture, MFLUCC 24-0239 • ibid., on stem of okra (*Abelmoschusesculentus*), February 2023, Maryam Fallahi, dried culture MF148 (MFLU 24-0238), living culture, MFLUCC 24-0256.

#### Notes.

**Figure 1.**
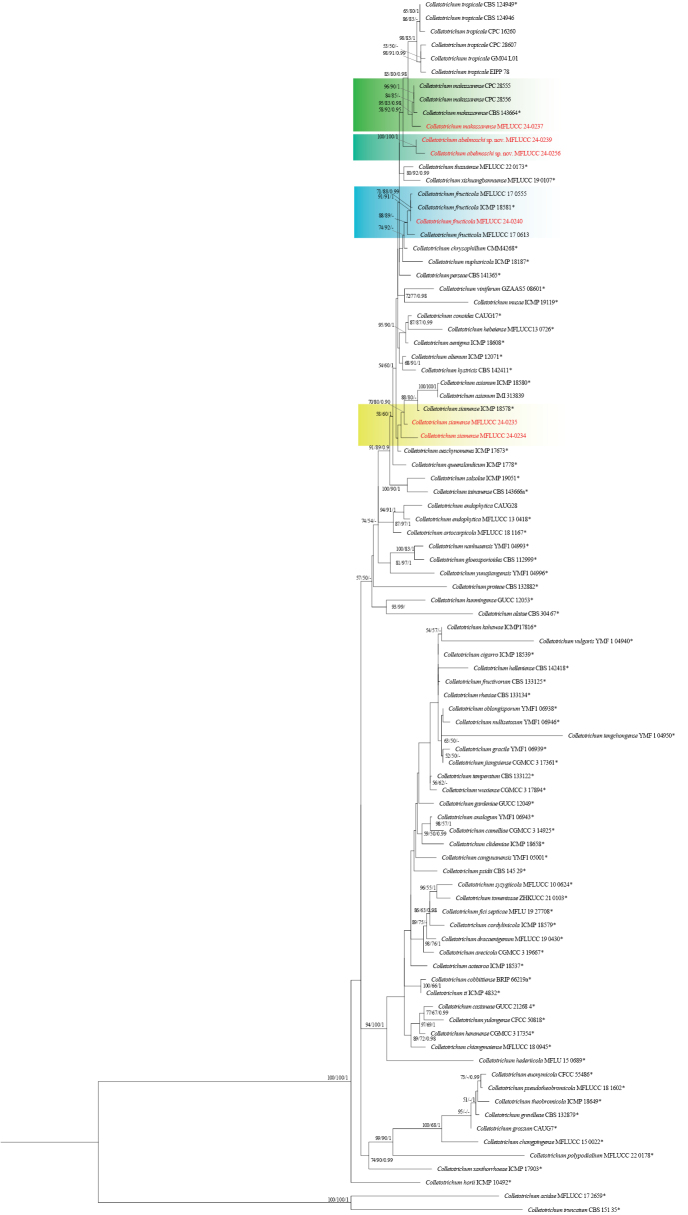
Phylogenetic tree of the *Colletotrichumgloeosporioides* species complex generated by maximum likelihood of combined ITS, *gapdh*, *chs-1*, *act*, and *tub2* sequence data. The ultrafast maximum likelihood (ML) and maximum parsimony (MP) bootstrap support values ≥50% (BT) as well as bayesian posterior probabilities ≥0.90 (BYPP) are shown, respectively, near the nodes. The ex-type strains are marked with an asterisk. The tree is rooted in *Colletotrichumacidae* (MFLUCC 17 2659) and *Colletotrichumtruncatum* (CBS 151 35).

Phylogenetic analyses showed that strains MFLUCC 24-0239 and MFLUCC 24-0256 form a distinct subclade in the *Colletotrichumgloeosporioides* species complex, basal to the subclade containing *C.tropicale* and *C.makassarense* with 58% ML, 92% MP bootstrap support, and 0.95 BYPP (Fig. 1) and are introduced as a new species, namely *Colletotrichumabelmoschi*. The base pair differences between *C.abelmoschi* (MFLUCC 24-0239, holotype), *C.tropicale* (CBS 124949, holotype), and *C.makassarense* (CBS 143664, ex-type) are presented in Table 2. Based on the morphology mentioned in Table 3, *Colletotrichumabelmoschi* (MFLUCC 24-0239) is different from *C.tropicale* (CBS 124949, holotype) and *C.makassarense* (CBS 143664) (Rojas et al. 2010; de Silva et al. 2019). *Colletotrichumabelmoschi* produced slightly narrower conidia than *C.tropicale* (CBS 124949) with an L/W ratio = 2.4 (8.6–13.7 × 4.2–5.8 μm in *Colletotrichumabelmoschi* vs. 10.2–12.7 × 8.2–11.2 μm in *C.tropicale* (CBS 124949)). Moreover, in comparison to *C.makassarense*, *Colletotrichumabelmoschi* produced slightly shorter conidia (8.6–13.7 × 4.2–5.8 μm in *Colletotrichumabelmoschi* (MFLUCC 24-0239) vs. 13–15 × 4.5–5 μm in *C.makassarense* (CBS 143664)) and conidiogenous cells (6–18 × 2.2–4 μm in *Colletotrichumabelmoschi* (MFLUCC 24-0239) vs. 7–25 × 3–4 μm in *C.makassarense* (CBS 143664)). There are limited reports on *Colletotrichum* species associated with okra. *Colletotrichumplurivorum* was identified as the primary causal agent of anthracnose on okra in Brazil (Batista et al. 2020). Shi et al. (2019) reported the presence of *C.gloeosporioides* causing anthracnose disease on okra in China (Shi et al. 2019). Based on morphology and multi-gene analysis, *Colletotrichumabelmoschi* is introduced as a new species on okra.

**Table 2.** The base pair differences between *Colletotrichumabelmoschi* (MFLUCC 24-0239, holotype), *Colletotrichumtropicale* (CBS 124949, holotype), and *Colletotrichummakassarense* (CBS 143664, ex-type).

	Gene region	*C.tropicale* (CBS 124949, holotype)	*C.makassarense* (CBS 143664, ex-type)
***Colletotrichumabelmoschi* (MFLUCC 24-0239, holotype)**	ITS	0.20% (1/502)	0.20% (1/502)
	*gapdh*	1.39% (3/215)	2.32% (5/215)
	*chs-1*	0.90% (2/220)	1.7%% (3/220)
	*act*	0.97% (2/206)	0.50% (1/206)
	*tub2*	0.73% (3/410)	1.5% (6/410)

**Table 3.** Characteristics of *Colletotrichumabelmoschi* (MFLUCC 24-0239) and the type strain of *Colletotrichumtropicale* (CBS 124949, Holotype) and *Colletotrichummakassarense* (CBS 143664, ex-type).

	Conidiogenous cells	Conidia	Appressoria	Setae	Culture characteristics
***C.abelmoschi* (MFLUCC 24-0239)**	cylindrical or doliiform, unbranched, guttulate, 6–18 × 2.2–4 μm	aseptate, straight, cylindrical, the apex and base rounded or tapered at one end, guttulate, 8.6–13.7 × 4.2–5.8 μm	ovoid, clavate, regular shape, or slightly irregular, brown to dark brown 4–12 × 5–7 μm	absent	60 mm after 7 d, circular shape, dull surface, entire edge, well-defined margin, with medium density, white to light grey
***C.tropicale* (CBS 124949)**	cylindrical, monoblastic, tip with periclinal thickening, arising from a thin base of textura epidermoidea, 7–15 × 3.5–4.5 μm.	subcylindrical with rounded ends, rarely clavate, straight, with or without a slightly protuberant, flat basal abscission scar, 10.2–12.7 × 8.2–11.2 μm.	subglobose, clavate, fusiform; not lobed, terminal, 7.0–11.0 × 5.2–7.2 μm	rare	40–50 mm after 4 d, white to light grey, no diffusing pigment, conidiomata forming abundantly in concentric rings, conidial masses slimy, orange.
***C.makassarense* (CBS 143664)**	subcylindrical, hyaline, smooth, phialidic with periclinal thickening, 7–25 × 3–4 μm.	hyaline, smooth, aseptate, subcylindrical, straight, apex obtuse, tapering at base to protruding truncate hilum, guttulate, 13–15× 4.5–5 μm.	subglobose, ellipsoidal, obovate, entire margin, 6–10.5 × 4–8.5 μm	present	45 mm after 7 d, surface smoke-grey, reverse olivaceous grey.

**Figure 2.**
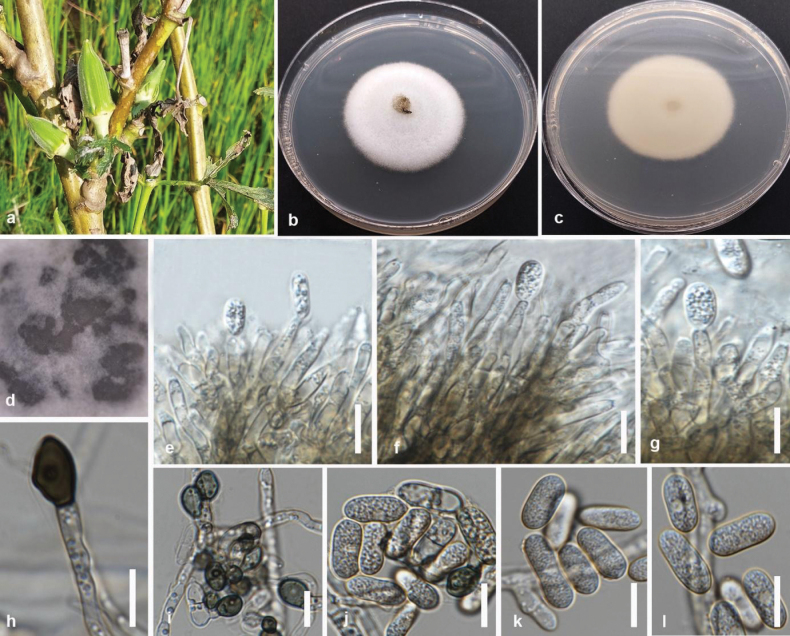
*Colletotrichumabelmoschi* (MFLUCC 24-0239, holotype) **a** leaf and stem blight on okra **b** front and **c** back view of the colony on PDA after 5 days **d** acervuli on PDA**e–g** conidiogenous cell **h, i** appressoria **j–l** conidia. Scale bars: 10 μm.

### ﻿ Colletotrichum fructicola

Taxon classificationFungiSordariomycetesGlomerellaceae

Prihast., L. Cai & K.D. Hyde, Fungal Diversity 39: 96 (2009)

044C302D-C094-5275-8395-9218F07F7627

Index Fungorum: IF515409

Facesoffungi Number: FoF06767

Fig. 3

#### Description.

Pathogenic to jackfruit (*Artocarpusheterophyllus*) and causes brown leaf spots and anthracnose on the foliage. Sexual morph not observed. Acervulus semi-immersed. Conidiophores reduced to conidiogenous cells. Conidia hyaline, cylindrical, with rounded apices, 9–15 × 3–6 µm (mean = 13 × 4.6 μm, n = 40). Appressoria brown to dark brown, clavate, ovoid, and slightly irregular or regular in shape, 8–10 × 4–8 µm (mean = 8 × 4.5 μm, n = 20). Chlamydospores and Setae are absent.

**Figure 3.**
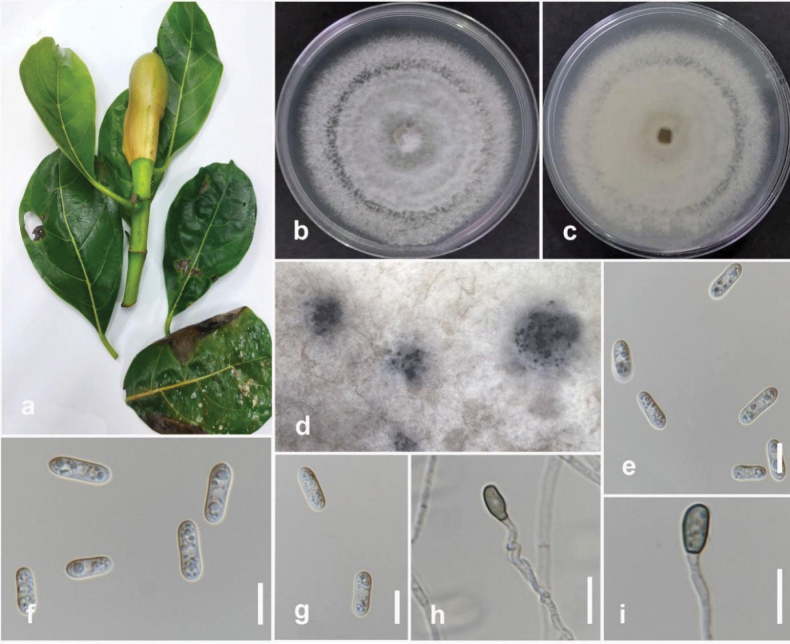
*Colletotrichumfructicola* (MFLUCC 24-0240) **a** brown leaf spot on jackfruit **b** front, and **c** back view of the colony on PDA after 7 days **d** acervuli on PDA**e–g** conidia **h, i** appressoria. Scale bars: 10 μm.

#### Culture characteristics.

Colonies on PDA reach 75 mm in diameter after 7 days of growth at 25 °C in the dark, cottony and circular with a dull surface and well-defined margin with medium density. The upper side is pale olivaceous grey in the center, smoke grey in the middle, and white at the margin. The reverse side shows circles of dull green with a greenish-grey color and a primrose margin.

#### Material examined.

Thailand • Chiang Rai, Phan District, Sai Khao, on the leaf of jackfruit (*Artocarpusheterophyllus*), January 2023, Maryam Fallahi, dried culture MF57-3 (MFLU 24-0241), living culture MFLUCC 24-0240.

#### Notes.

Based on phylogenetic analysis, strain MFLUCC 24-0240 clustered in the same subclade as *Colletotrichumfructicola* (ICMP 18581, ex-type) with 91% ML, 91% MP bootstrap support, and 1.0 BYPP (Fig. 1). The base pair differences between *C.fructicola* (ICMP 18581, ex-type) and *C.fructicola* (MFLUCC 24-0240) revealed no differences in ITS, *gapdh*, *chs-1*, *act*, and *tub2*. *Colletotrichumfructicola* (MFLUCC 24-0240) is similar to *C.fructicola* (MFLU 090228, holotype), although it produces slightly wider conidia with an L/W ratio of 2.8 (9–15 × 3–6 µm in *C.fructicola* (MFLUCC 24-0240) vs. 9.7–14 × 3–4.3 μm in *C.fructicola* (MFLU 090228)) (Prihastuti et al. 2009). *Colletotrichumfructicola* has an extensive host range and geographical distribution (Weir et al. 2012; Norphanphoun and Hyde 2023). It was originally reported from coffee berries in Thailand (Prihastuti et al. 2009) and causes anthracnose, bitter rot, and leaf-spotting diseases in a wide range of woody or herbaceous plants (Bragard et al. 2021). *Colletotrichumfructicola* is associated with jackfruit anthracnose worldwide, especially in Thailand (Borges et al. 2023). *Colletotrichumartocarpicola* and *C.gloeosporioides* have been reported from jackfruit in northern Thailand (Bhunjun et al. 2019). In this study, *C.fructicola* was isolated from jackfruit and described as a new host record in northern Thailand.

### ﻿ Colletotrichum makassarense

Taxon classificationFungiSordariomycetesGlomerellaceae

D.D. de Silva, Crous & P.W.J. Taylor, IMA Fungus 10: 23 (2019)

7F9182B2-310B-5F0D-BBA3-2CBE93F2775C

Index Fungorum: IF827691

Fig. 4

#### Description.

Pathogenic to tamarind (*Tamarindusindica*) and causes brown leaf spots on leaves. Sexual morph not observed. Conidiomata acervular, forming abundantly on CLA media, producing orange conidial masses. Conidiophores subcylindrical or subcylindrical, septate, hyaline, smooth, branched. Conidiogenous cells cylindrical, branched, monoblastic, and 7–30 × 1.5–3 μm. Conidia straight, guttulate, subcylindrical, hyaline, aseptate, apex rounded and slightly tapering at the base, 12–14.5 × 4–5.4 μm (mean = 13 × 4.7 μm, n = 40). Appressoria of diverse shape, lobate, brown to dark brown, irregular margin, and 5–12.5 × 4–10 μm (mean = 8 × 7 μm, n = 20). Chlamydospores and setae are absent.

**Figure 4.**
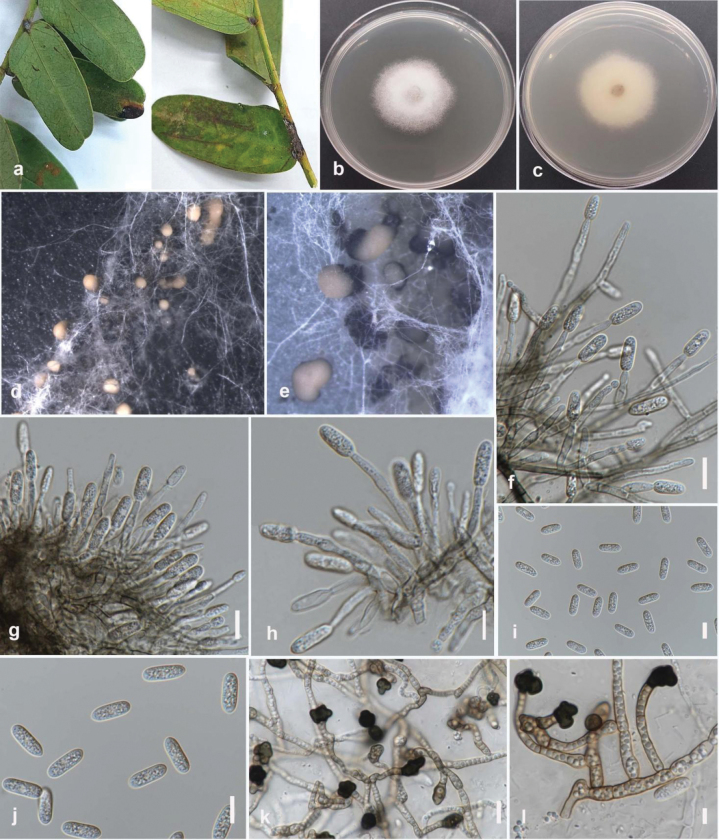
*Colletotrichummakassarense* (MFLUCC 24-0237) **a** brown leaf spot on tamarind **b** front, and **c** back view of the colony on PDA after 4 days **d–e** acervuli on CLA **f–h** conidiophores and conidiogenous cells **i, j** conidia **k, l** appressoria Scale bars: 10 μm (**g–k**); 5 μm (**l**).

#### Culture characteristics.

Colonies on PDA reach 60 mm in diameter after 7 days of growth at 25 °C in the dark, fluffy to cottony; circular shape, entire edge, with fluffy margin and medium density. Upper view white to smoke grey and reverse primrose.

#### Material examined.

Thailand • Chiang Rai Province, Phan District, Sai Khao, on tamarind (*Tamarindusindica*), February 2023, Maryam Fallahi, dried culture MF99-3 (MFLU 24-0235), living culture, MFLUCC 24-0237.

#### Notes.

In the phylogenetic tree generated in this study, strain MFLUCC 24-0237 clustered with strains of *Colletotrichummakassarense* by 95% ML, 83% MP bootstrap support, and 0.98 BYPP (Fig. 1). The base pair differences between *C.makassarense* strains CBS 143664 (ex-type) and MFLUCC 24-0237 revealed 0.21% (1/475) differences in ITS, 1.42% (3/211) differences in *gapdh*, 1.26% (3/237) differences in *chs-1*, 0.24% (1/410) differences in *tub*, and no difference in *act. Colletotrichummakassarense* (MFLUCC 24-0237) shows some difference to *C.makassarense* (CBS 143664, ex-type) by producing conidiogenous cells with an L/W ratio = 11 (7–30 × 1.5–3 μm in *C.makassarense* (MFLUCC 24-0237) vs. 7–25 × 3–4 μm in *C.makassarense* (CBS 143664, ex-type)). *Colletotrichummakassarense* (MFLUCC 24-0237) produced appressoria with diverse shapes and sizes (lobate, brown to dark brown, irregular margin, and 5–12.5 × 4–10 in *C.makassarense* (MFLUCC 24-0237) vs. solitary, medium brown, smooth-walled, subglobose, ellipsoidal to obovate, entire margin, 6–10.5 × 4–8.5 μm in *C.makassarense* (CBS 143664, ex-type)), and setae were absent in *C.makassarense* (MFLUCC 24-0237); however, *C.makassarense* (CBS 143664, ex-type) produced medium brown setae (de Silva et al. 2019). Also, *C.makassarense* (MFLUCC 24-0237) showed a high growth rate (60 mm after seven days on PDA) compared to *C.makassarense* (CBS 143664, ex-type) (40 mm after seven days on PDA). *Colletotrichummakassarense* is phylogenetically closely related to *C.tropicale*; however, they are distinguished by multi-phylogenetic analysis (de Silva et al. 2019). It was first isolated from fruit lesions of *Capsicumannuum* in Indonesia (de Silva et al. 2019). This study provided the first report of *C.makassarense* in tamarind (*Tamarindusindica*).

### ﻿ Colletotrichum siamense

Taxon classificationFungiSordariomycetesGlomerellaceae

Prihast., L. Cai & K.D. Hyde, Fungal Diversity 39: 98 (2009)

AE9ADE2C-030C-5003-AF1E-7177F6C8B83C

Index Fungorum: IF515410

Facesoffungi Number: FoF03599

Fig. 5

#### Description.

Pathogenic to black pepper (*Pipernigrum*) and causes brown leaf spots on leaves. Sexual morph not observed. Conidiophores hyaline, branched, or unbranched. Conidiogenous cells hyaline, cylindrical 7–20 × 1–2 μm (mean = 14 × 1.5 μm, n = 15). Conidia hyaline, fusiform, obtuse to slightly rounded at the ends, and sometimes oblong, single-celled, smooth-walled, guttulate, 8–18.3 × 3–5 μm (mean = 15 × 4.3 μm, n = 40). Appressoria frequently form from mycelia in slide cultures, brown, ovoid, sometimes clavate, and turn complex with age, 5–10 × 4.5–6.5 μm (mean = 8 × 5.5 μm, n = 20). Chlamydospores and Setae are absent.

**Figure 5.**
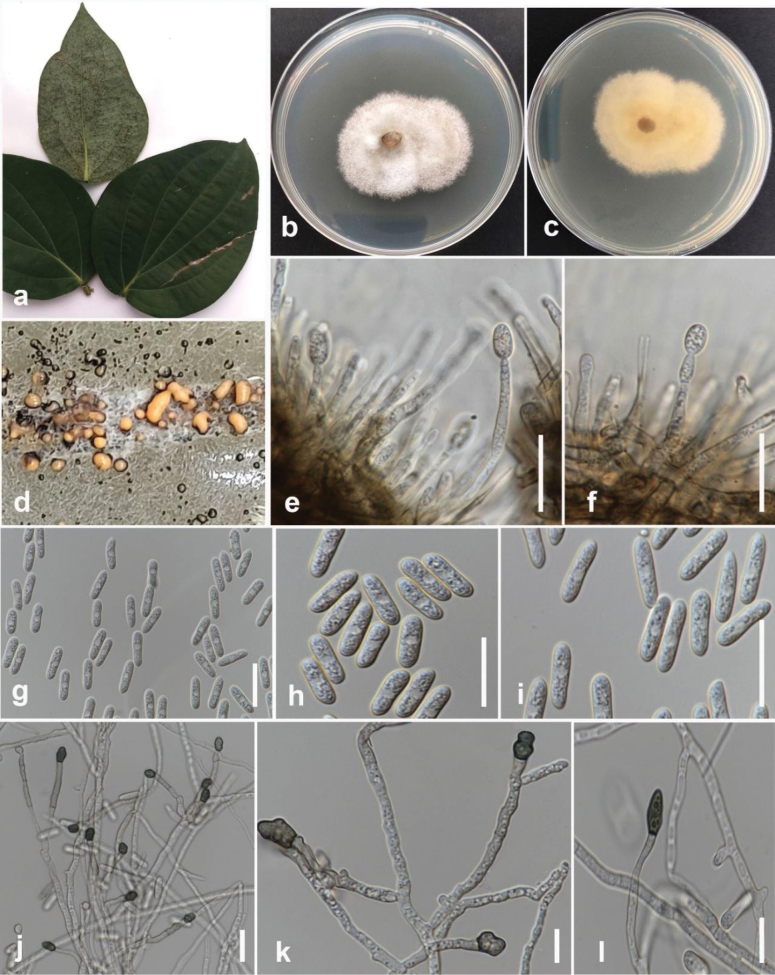
*Colletotrichumsiamense* (MFLUCC 24-0235) **a** leaf of black pepper **b** front, and **c** back view of the colony on PDA after five days **d** acervuli on CLA **e, f** conidiophores and conidiogenous cells **g–i** conidia **j–l** appressoria. Scale bars: 20 μm (**e–j**); 10 μm (**k–l**).

#### Culture characteristics.

Colonies on PDA reach 70 mm in diameter after 7 days of growth at 25 °C in the dark, cottony, with medium density. The colony’s surface is covered with numerous small acervuli, with orange conidial ooze. The upper view is white to light grey, and the reverse is greyish to pale yellowish.

#### Material examined.

Thailand • Chiang Rai Province, Phan District, Sai Khao. On a leaf of black pepper (*Pipernigrum*), December 2022, Maryam Fallahi, dried culture MF21-4 (MFLU 24-0240), living culture, MFLUCC 24-0235 • Chiang Rai Province, Phan District, Sai Khao, on Brazil cherry (*Eugeniabrasiliensis*), December 2022, Maryam Fallahi, dried culture MF17-4 (MFLU 24-0239), living culture, MFLUCC 24-0234.

#### Notes.

On the phylogenetic tree, strains MFLUCC 24-0235 and MFLUCC 24-0234 were placed close to *Colletotrichumsiamense* (ICMP 18578, ex-type) (Fig. 1). The base pair differences between *C.siamense* strains ICMP 18578 and MFLUCC 24-0235 revealed 0.9% (2/222 bp) differences in *chs-1*, 0.97% (2/206 bp) differences in *act*, 1.3% (5/366 bp) differences in *tub2*, and no difference in *gapdh* and ITS. Strain MFLUCC 24-0235 is similar to *C.siamense* (MFLU 090230, holotype) (Prihastuti et al. 2009). However, it produced slightly larger conidia with an L/W ratio = 3.5 (8–18.3 × 3–5 μm in *C.siamense* (MFLUCC 24-0235) vs. 7–18.3 × 3–4.3 μm (L/W ratio = 5) in *C.siamense* (MFLU 090230, holotype)) and larger appressoria (5–10 × 4.5–6.5 μm (L/W ratio = 1.4) in *C.siamense* (MFLUCC 24-0235) vs. 4.7–8.3 × 3.5–5 μm (L/W ratio = 1.6) in *C.siamense* (MFLU 090230)) (Prihastuti et al. 2009). Phylogenetically, *C.siamense* is closely related to *C.asianum*; however, morphologically, they are different (Prihastuti et al. 2009). *Colletotrichumsiamense* has been reported as a pathogen on many host plants (Than et al. 2008; Talhinhas and Baroncelli 2021, 2023). It was isolated from anthracnose leaf spot on black pepper in northeast India (Verma et al. 2023). To our knowledge, this is the first report of *C.siamense* on black pepper in Thailand.

### ﻿ Colletotrichum plurivorum

Taxon classificationFungiSordariomycetesGlomerellaceae

Damm, Alizadeh & Toy, Stud. Mycol. 92: 1–46. 2019

A2CC8F88-F43B-50CE-A365-BF5D0B76151F

Index Fungorum: IF824228

Facesoffungi Number: FoF10691

Fig. 7

#### Description.

Pathogenic to persimmon (*Diospyrosehretioides*) and causes brown leaf spots on leaves. Sexual morph: Ascomata perithecial, solitary, superficial or immersed, non-stromatic, globose to pyriform, ostiolate, glabrous. Asci unitunicate, 8-spored, cylindrical, smooth-walled, broadly truncated at the base. Ascospores uni- or biseriate, aseptate, hyaline, pale brown with age, allantoid to fusiform, rounded ends, smooth-walled. Asexual morph: conidiophores pale brown, smooth-walled, simple or septate, branched, up to 30 μm long. Conidiogenous cells pale brown, smooth-walled, cylindrical, 7–19 × 4–5.5 μm. Conidia hyaline, smooth-walled, aseptate, straight, cylindrical, sometimes slightly clavate on apex, base rounded, 10–20 × 4–8 μm (mean = 13 × 5 μm, n = 30). Appressoria solitary, dark brown, irregular in outline, undulate to lobate margin, 6–8 × 5.5–8 μm (mean = 7 × 6.5 μm, n = 20). Chlamydospores and Setae are absent.

#### Culture characteristics.

Colonies on PDA reach 60 mm in diameter after 7 days of growth at 25 °C in the dark, flat with medium density, entire margin fluffy. The upper view is olivaceous grey in the center and greyish-white in the margin. The reverse is olivaceous in the center with olivaceous and primrose circles.

#### Material examined.

Thailand • Chiang Rai Province, Mueang Chiang Rai District, Doi Hang, on persimmon (*Diospyrosehretioides*), February 2023, Maryam Fallahi, dried culture MF117-1 (MFLU 24-0236), living culture, MFLUCC 24-0238.

#### Notes.

**Figure 6.**
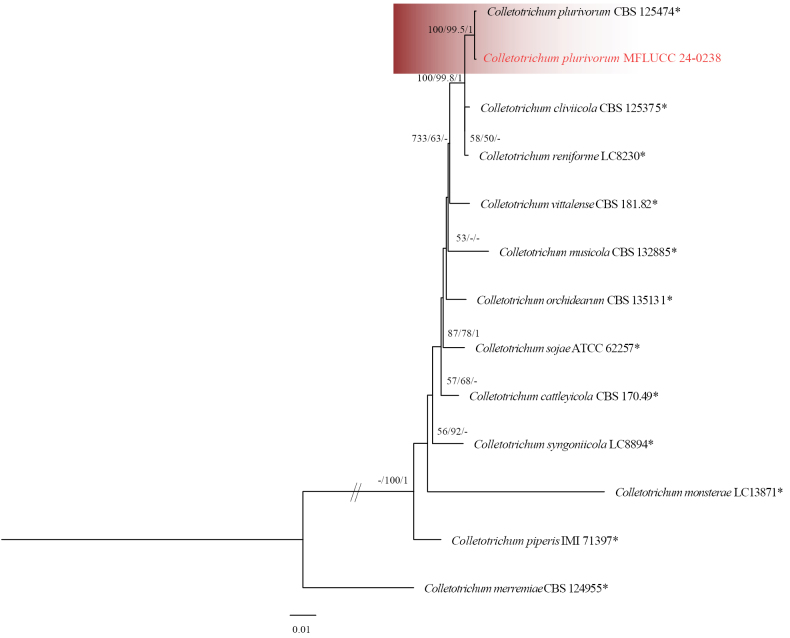
Phylogenetic tree of the *Colletotrichumorchidearum* species complex generated by maximum likelihood of combined ITS, *gapdh*, *chs-1*, *act*, and *tub2* sequence data. The ultrafast maximum likelihood (ML) and maximum parsimony (MP) bootstrap support values ≥50% (BT) as well as Bayesian posterior probabilities ≥0.90 (BYPP) are shown, respectively, near the nodes. The ex-type strains are marked with an asterisk. The tree is rooted in *Colletotrichummerremiae* (CBS 124955).

Strain MFLUCC 24-0238 clustered with *Colletotrichumplurivorum* (CBS 125474, ex-holotype) in the *Colletotrichumorchidearum* species complex by 100% ML, 100% MP bootstrap support, and 1.0 BYPP (Fig. 6). The base pair differences between *C.plurivorum* strains MFLUCC 24-0238 and CBS 125474 revealed 0.53% (1/187 bp) differences in *gapdh*, 0.46% (1/217 bp) differences in *act*, and no difference in ITS, *chs-1*, and *tub2. Colletotrichumplurivorum* (MFLUCC 24-0238) is similar to *C.plurivorum* in morphology (CBS 125474) (Damm et al. 2019). However, it produced slightly shorter appressoria with an L/W ratio = 1.1 (6–8 × 5.5–8 μm in *C.plurivorum* (MFLUCC 24-0238) vs. 12.5–18.5 × 6.5–11.5 μm (L/W ratio = 1.75) in *C.plurivorum* (CBS 125474, ex-type)) (Damm et al. 2019). *Colletotrichumplurivorum* is closely related to *C.cliviicola* and differs by a few nucleotides in each gene. The asexual morph of the two species is morphologically similar. However, the growth rate of *C.plurivorum* is slower than that of *C.cliviicola* (Damm et al. 2009). *Colletotrichumplurivorum* was first isolated from *Capsicumannuum* in the Sichuan province of China and has a wide host range, including *Phaseoluslunatus*, P. vulgaris, *Gossypium* spp., *Spathiphyllumwallisii*, and *Coffea* spp. (Damm et al. 2019; Hassan et al. 2022; Liu et al. 2016). In this study, we isolated *C.plurivorum* from persimmon in northern Thailand as a new host record.

**Figure 7.**
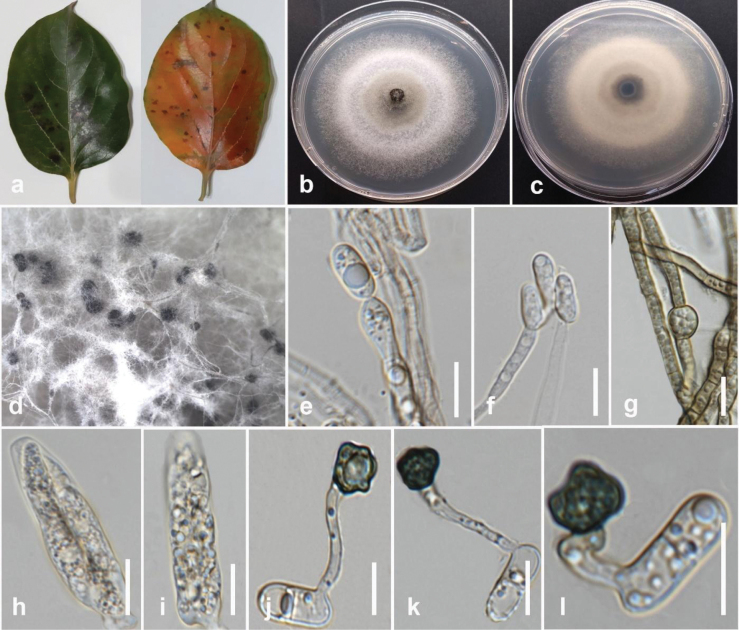
*Colletotrichumplurivorum* (MFLUCC 24-0238) **a** brown leaf spot on persimmon leaves **b** front, and **c** back view of the colony on PDA after seven days **d** acervuli on PDA**e, f** conidiophores and conidiogenous cells **g** chlamydospore **h, i** ascus **j–l** appressoria. Scale bars: 10 μm.

### ﻿ Colletotrichum spaethianum

Taxon classificationFungiSordariomycetesGlomerellaceae

(Allesch.) Damm, P.F. Cannon & Crous, Fungal Diversity 39: 74 (2009)

1CE63D5B-0F7A-5DDB-9B61-F668CD207574

Index Fungorum: IF514644

Facesoffungi Number: FoF05784

Fig. 9

#### Description.

Associated with brown leaf spot of edible canna (*Cannaindica*). Sexual morph not observed. Conidiomata acervulus, immersed or semi-immersed. Conidiophores formed directly on hyphae, hyaline, septate, and branched, up to 60 μm long. Conidiogenous cells enteroblastic, hyaline, cylindrical, slightly inflated, 8–18 × 2–3 μm. Conidia hyaline, smooth-walled, aseptate, slightly curved, 13–22 × 3.5–4 μm (mean = 17 × 3.6 μm, n = 20). Appressoria single or in loose groups, dark brown, irregular in shape, sometimes slightly lobed, smooth-walled 7–19 × 5–7.5 μm (mean = 13 × 6 μm, n = 30). Chlamydospores and Setae are absent.

#### Culture characteristics.

Colonies on PDA reach 60 mm in diameter after 7 days of growth at 25 °C in the dark, circular, with dull surfaces and entire margins. The upper view is cottony with medium density, greyish-white in the center, flat, fluffy, and pinkish-white in other parts. The reverse has dark grey to orange pigmentation in the center and primrose in other parts.

#### Material examined.

Thailand • Muang Chiang Mai, Mushroom Research Center, on edible canna (*Cannaindica*), 19 February 2023, Maryam Fallahi, dried culture MF140-1 (MFLU 24-0242), living culture, MFLUCC 24-0236.

#### Notes.

**Figure 8.**
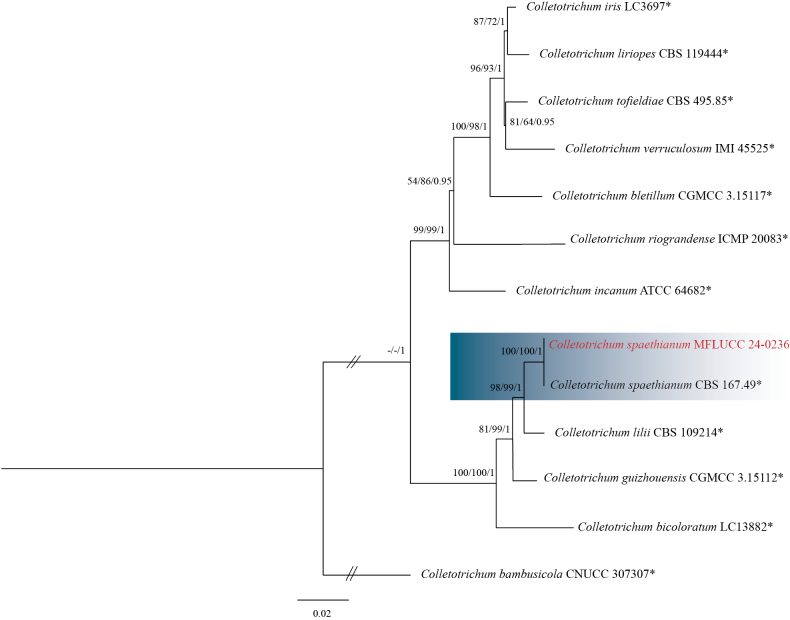
Phylogenetic tree of the *Colletotrichumspaethianum* species complex generated by maximum likelihood of combined ITS, *gapdh*, *chs-1*, *act*, and *tub2* sequence data. The ultrafast maximum likelihood (ML) and maximum parsimony (MP) bootstrap support values ≥50% (BT) as well as Bayesian posterior probabilities ≥0.90 (BYPP) are shown, respectively, near the nodes. The ex-type strains are marked with an asterisk. The tree is rooted in *Colletotrichumbambusicola* (CNUCC 307307).

Based on the phylogenetic tree generated in this study, strain MFLUCC 24-0236 grouped with *Colletotrichumspaethianum* (CBS 167-49, ex-epitype) in the *C.spaethianum* species complex by 100% ML, 100% MP bootstrap support, and 1.0 BYPP (Fig. 8). The base pair differences between *C.spaethianum* strains MFLUCC 24-0236 and CBS 167-49 revealed no difference in ITS, *gapdh*, *chs-1*, *act*, and *tub2*. *Colletotrichumspaethianum* (MFLUCC 24-0236) is similar to *C.spaethianum* (CBS 167-49). However, it produced slightly larger conidiogenous cells with an L/W ratio = 6 (8–18 × 2–3 μm in *C.spaethianum* (MFLUCC 24-0236) vs. 6–16 × 3–4 μm (L/W ratio = 5.4) in *C.spaethianum* (CBS 167–49)) and bigger appressoria (7–19 × 5–7.5 μm (L/W ratio = 2.2) in *C.spaethianum* (MFLUCC 24-0236) vs. 7–9.5 × 5–7.5 μm (L/W ratio = 1.3) in *C.spaethianum* (CBS 167-49)) (Damm et al. 2009). Also, unlike *C.spaethianum* (CBS 167-49), setae were not observed in *C.spaethianum* (MFLUCC 24-0236) (Damm et al. 2009). In our phylogenetic analysis, *C.spaethianum* (CBS 167-49) clustered in a well-supported subclade with *C.guizhouensis* (CGMCC 3.15112, ex-type), *C.lilii* (CBS 109214), and *C.bicoloratum* (NN055229). *Colletotrichumspaethianum* is similar to the other three species in conidial shape and differs in setae, which usually have an acute tip and a cylindrical to conical base in C. *spaethianum*. The appressoria of *C.spaethianum* have an irregular outline that is more or less lobed (Damm et al. 2009). *Colletotrichumspaethianum* was first reported on dead stems of *Funkiaunivittata* from Germany (Damm et al. 2009). It usually causes leaf spots and anthracnose on various hosts (Liu et al. 2020; Ma et al. 2020; Sun et al. 2020). This study provided the first host record for *C.spaethianum* on edible canna in Thailand.

**Figure 9.**
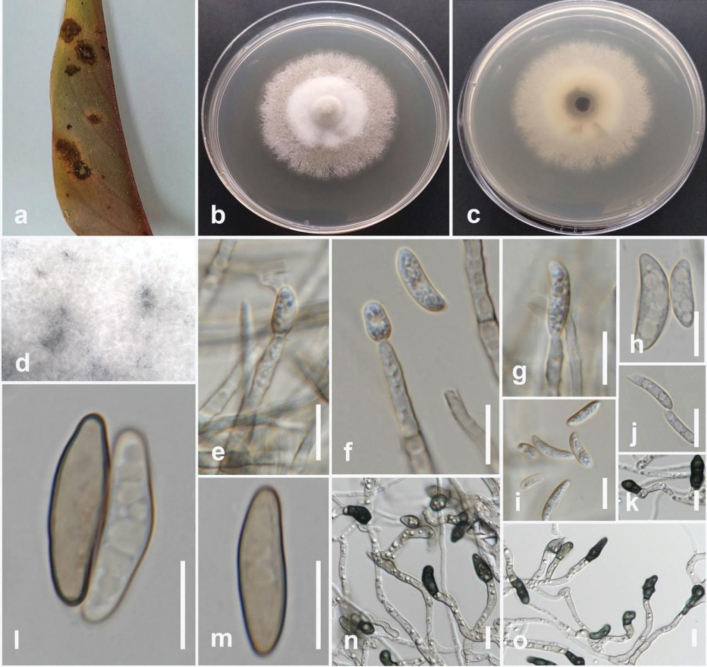
*Colletotrichumspaethianum* (MFLUCC 24-0236) **a** brown leaf spot in edible canna **b** front and **c** back view of the colony on PDA after seven days **d** acervuli on PDA**e–g** conidiophores and conidiogenous cells **h–j, l, m** conidia **k, n, o** appressoria. Scale bars: 10 μm.

### ﻿ Diaporthe

Taxon classificationFungiSordariomycetesDiaporthaceae

Nitschke, Pyrenomycetes Germanici. 2: 240 (1870)

75CF43E5-B30A-598E-87B2-3614276B0D3C

#### Notes.

In the present study, based on phylogenetic analyses of the ITS, *tef1*, *tub2*, *cal*, and *his3* sequence data and morphology, four well-known species, viz., *Diaporthehongkongensis*, *D.rosae*, *D.sennicola*, and *D.siamensis*, and two novel species, viz., *Diaporthefistulosi* and *Diaporthemelongenicola*, are illustrated.

### ﻿ Diaporthe sennicola

Taxon classificationFungiSordariomycetesDiaporthaceae

C.M. Tian & Qin Yang, Phytotaxa 302(2): 150 (2017)

9414B57E-9A05-501F-BB15-C92009E75748

Index Fungorum: IF820453

Fig. 11

#### Description.

Pathogenic to okra (*Abelmoschusesculentus*) and causes brown stem lesion (canker) of the stem. Sexual morph not observed. Conidiomata pycnidial, immersed, scattered, circular, or ovoid in shape, with single, undivided loculus. Ectostromatic disc dark brown, flat, elliptical. Conidiophores hyaline, branched, phialidic, straight or partially curved, narrowing towards the apex, 11.5–17 × 1–1.6 μm (12.7 × 1.3 μm, n = 20). Conidiogenous cells hyaline, phialidic, straight, or partially curved. Alpha conidia hyaline, aseptate, elliptic, biguttulate, 5–9 × 1.3–2.7 μm (6.5 × 2.3 μm, n = 40). Beta and gamma conidia are absent.

#### Culture characteristics.

Colonies on PDA reach 50–55 mm in diameter after 7 days of growth at 25 °C in the dark, flat, and white, turning into greyish rose with age. Compact, furcate mycelium is erratically distributed over the agar surface, edge irregular .

#### Material examined.

Thailand • Sakon Nakhon Province, Mueang Sakon Nakhon District, on stem lesion of okra (*Abelmoschusesculentus*), February 2023, Maryam Fallahi, dried culture MF148-2 (MFLU 24-0243), living culture, MFLUCC 24-0241.

#### Notes.

**Figure 10.**
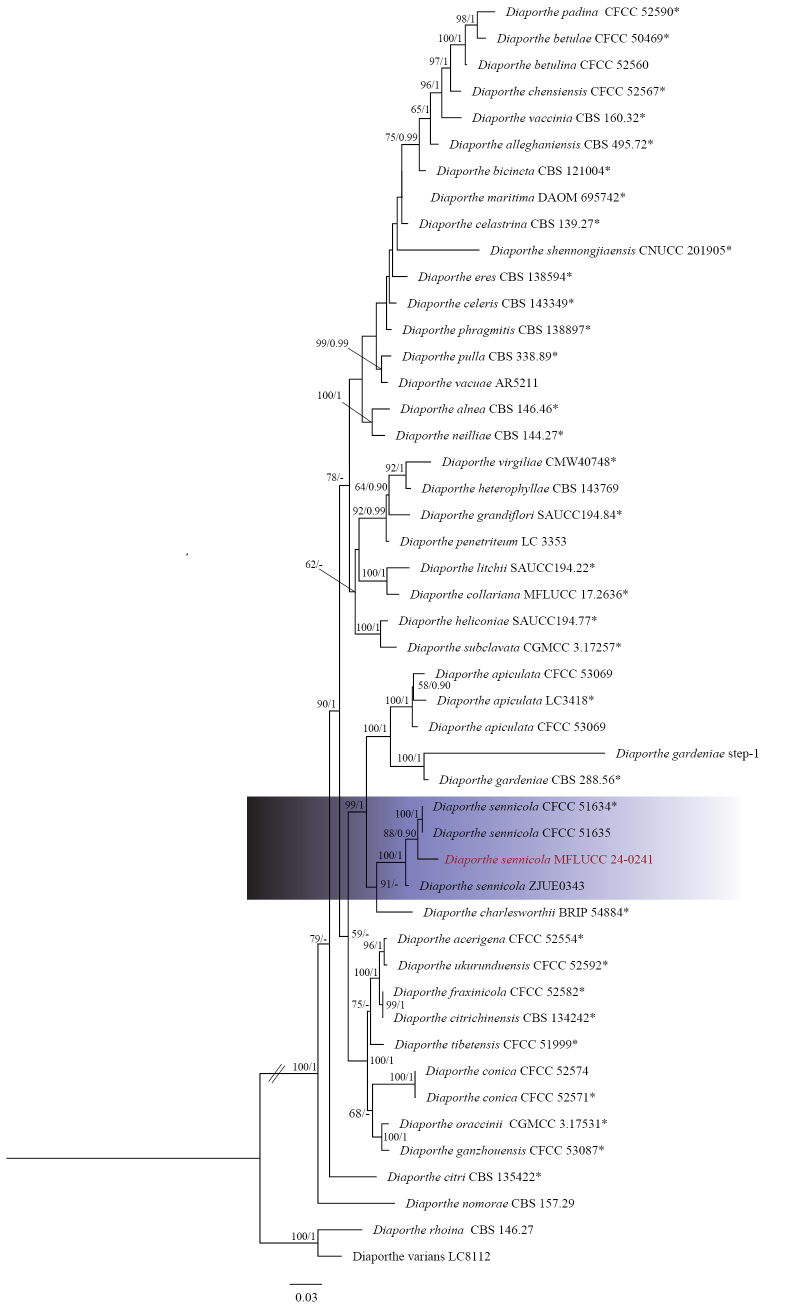
Phylogenetic tree of the *Diaporthealnea* species complex generated by maximum likelihood of combined ITS, *tef1*, *tub2*, *cal*, and *his3* sequence data. The ultrafast maximum likelihood (ML) bootstrap support values ≥50% (BT) and bayesian posterior probabilities ≥0.90 (BYPP) are shown, respectively, near the nodes. The ex-type strains are marked with an asterisk. The tree is rooted in *Diaportherhoina* (CBS 146.27) and *Diaporthevarians* (LC8112).

In the phylogenetic tree generated in this study, strain MFLUCC 24-0241 clustered with strains of *Diaporthesennicola* (CFCC 51634 (ex-type) and CFCC 51635) with 100% ML bootstrap support, and 1.0 BYPP (Fig. 10). The base pair differences between *D.sennicola* strains MFLUCC 24-0241 and ex-type CFCC 51634 revealed 2.4% (11/455 bp) differences in ITS, 2.9% (8/276 bp) differences in *tef1*, 2.8% (13/464 bp) differences in *tub2*, and 0.4% (2/455 bp) differences in *cal*. The sequence data of *his3* is not available for *D.sennicola* (CFCC 51634). *Diaporthesennicola* (MFLUCC 24-0241) is similar to *D.sennicola* (BJFC-S1368, holotype) (Yang et al. 2017). Based on phylogenetic analyses conducted by Norphanphoun et al. (2022), it was clustered in the *D.alnea* species complex. In the phylogenetic tree of Dong et al. (2020), *D.sennicola* is similar to *D.clausenae*. However, *D.clausenae* can be distinguished from *D.sennicola* by the presence of 2–4 large or small guttules in alpha conidia, as well as the presence of beta conidia (Hongsanan et al. 2023). This study identifies okra as a new host for *D.sennicola*.

**Figure 11.**
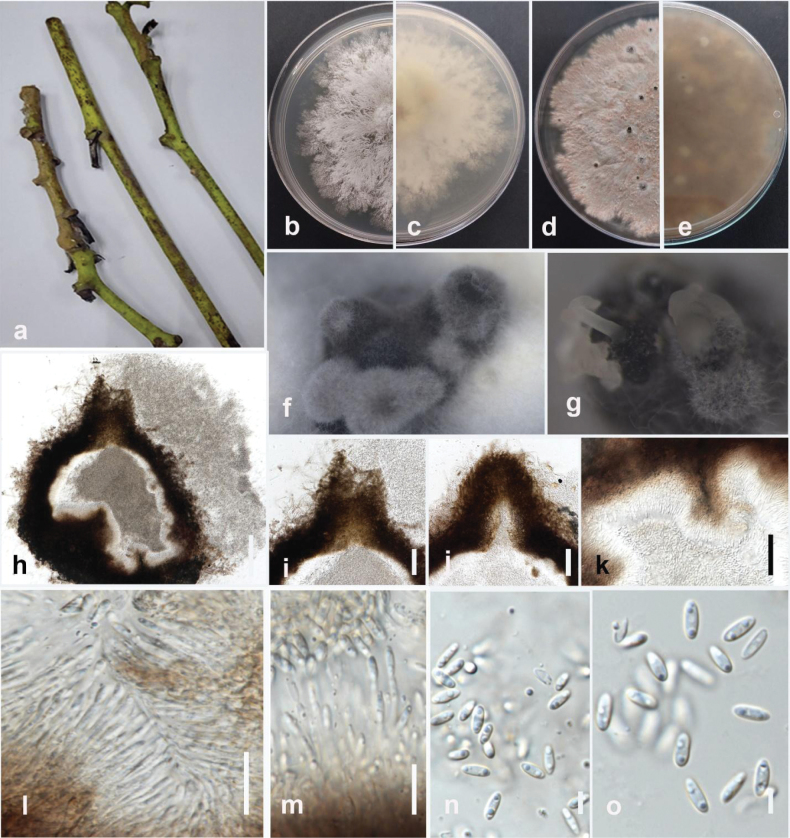
*Diaporthesennicola* (MFLUCC 24-0241) **a** brown stem lesion (canker) on okra **b** front and **c** back view of the colony on PDA after ten days **d** front and **e** back view of the colony on PDA after 60 days **f, g** conidiomata **h–j** section view of conidiomata **k, l** conidiophores **m, n** alpha conidia. Scale bars: 100 μm **(h–j)**; 50 μm **(k**); 20 μm **(l)**; 10 μm (**m**); 5 μm **(n, o)**.

### ﻿ Diaporthe fistulosi

Taxon classificationFungiSordariomycetesDiaporthaceae

Fallahi, Jayawar. & K.D. Hyde sp. nov.

1B075291-99B4-5A5E-98C6-DB33089DBCCA

Index Fungorum: IF903265

Fig. 13

#### Etymology.

‘*fistulosi*’ refers to the host plant species from which the fungus was isolated.

#### Description.

Pathogenic to spring onion (*Alliumfistulosum*) and causes basal rot and wilting symptoms on infected plants. Sexual morph not observed. Pycnidia immersed with black neck, slightly elongated. Conidiophores densely aggregated, unbranched, hyaline, subcylindrical, straight to sinuous, 6.5–17.5 × 1.5–2.5 μm (mean = 13 × 1.5 μm, n = 10). The conidiogenous cells phialidic, terminal, slightly tapering towards the apex. Paraphyses elongate above the conidiophores, hyaline, smooth, cylindrical, septate, unbranched, 10–20 × 2–2.5 μm (mean = 15 × 2.3 μm, n = 15). Beta conidia filiform, curved at one tip, hyaline, aseptate, rounded at tips, 15–25 × 1–1.4 μm (23 × 1 μm, n = 40). Alpha and gamma conidia are absent.

#### Culture characteristics.

Colonies on PDA reach 65–70 mm in diameter after 7 days of growth at 25 °C in the dark, covered with plenteous greyish-white villous aerial mycelium after 7 days. The reverse is slightly reddish-yellow in the center, with black fruiting bodies with age.

#### Material examined.

Thailand • Chiang Rai Province, Mueang Chiang Rai District, Doi Hang, spring onion (*Alliumfistulosum*), February 2023, Maryam Fallahi, dried culture MF112-3 (MFLU 24-0261, holotype), ex-holotype culture, MFLUCC 24-0244.

#### Notes.

**Figure 12.**
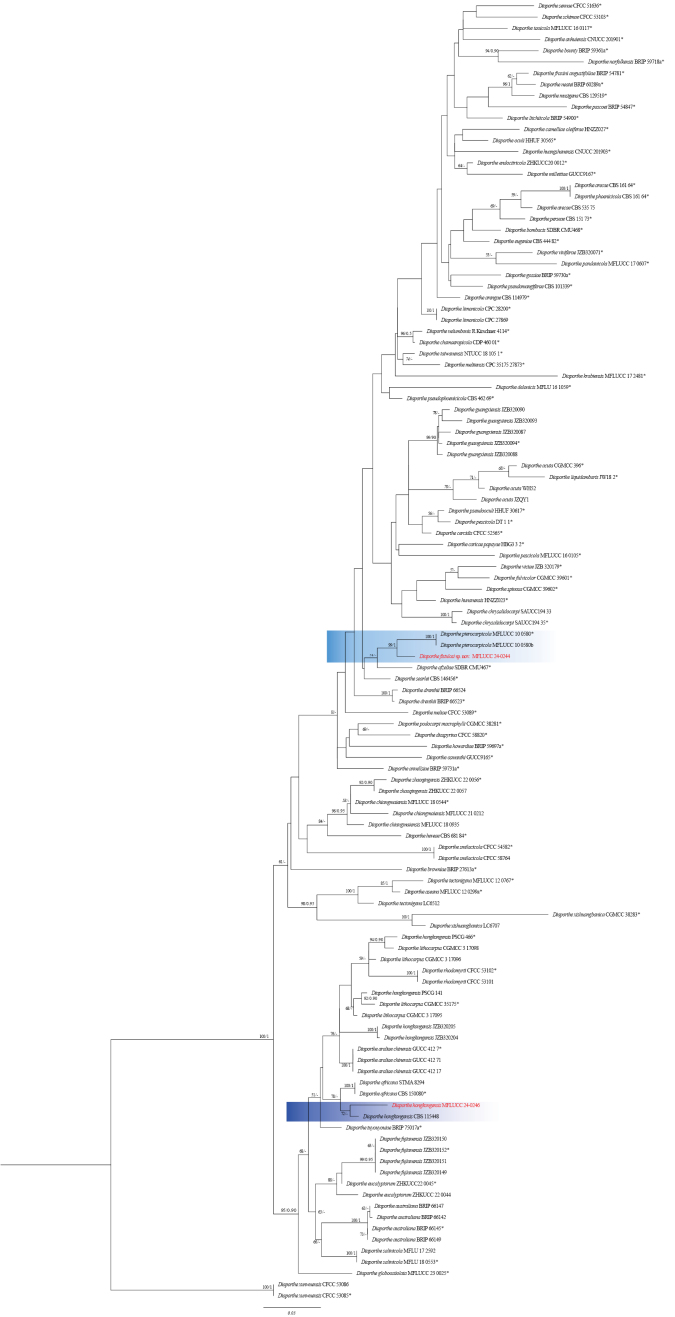
Phylogenetic tree of the *Diaporthearecae* species complex generated by maximum likelihood of combined ITS, *tef1*, *tub2*, *cal*, and *his3* sequence data. The ultrafast maximum likelihood (ML) bootstrap support values ≥50% (BT) and Bayesian posterior probabilities ≥0.90 (BYPP) are shown, respectively, near the nodes. The ex-type strains are marked with an asterisk. The tree is rooted in two strains of *Diaporthexunwuensis* (CFCC 53086, CFCC 53085).

Based on phylogenetic analysis of ITS, *tef1*, *tub2*, *cal*, and *his3* sequence data, the strain MFLUCC 24-0244 formed a distinct branch within the subclade containing *Diaporthepterocarpicola* (MFLUCC 10-0580a [ex-type] and MFLUCC 10-0580b) in the *Diaporthearecae* species complex, supported by 100% ML bootstrap and 1.0 BYPP (Fig. 12). It is introduced here as a new species, *Diaporthefistulosi*. The base pair differences between *D.fistulosi* strains MFLUCC 24-0244 (ex-holotype) and MFLUCC 10-0580a were 0.97% (5/513 bp) in ITS, 0.31% (1/315 bp) in *tef1*, 0.25% (1/391 bp) in *tub2*, and 1.4% (3/212 bp) in *cal*. The sequence data of *his3* is not available for *D.pterocarpicola* (MFLUCC 10-0580a). Unlike the holotype of *D.pterocarpicola* (MFLU 12-0129), which produced alpha conidia and did not produce beta conidia (Udayanga et al. 2012), *D.fistulosi* (MFLUCC 24-0244) only produced beta conidia. A pairwise homoplasy index (PHI) test indicated no significant recombination (Φw = 1.0) between *D.fistulosi* (MFLUCC 24-0244) and its closely related taxa (Fig. 14).

**Figure 13.**
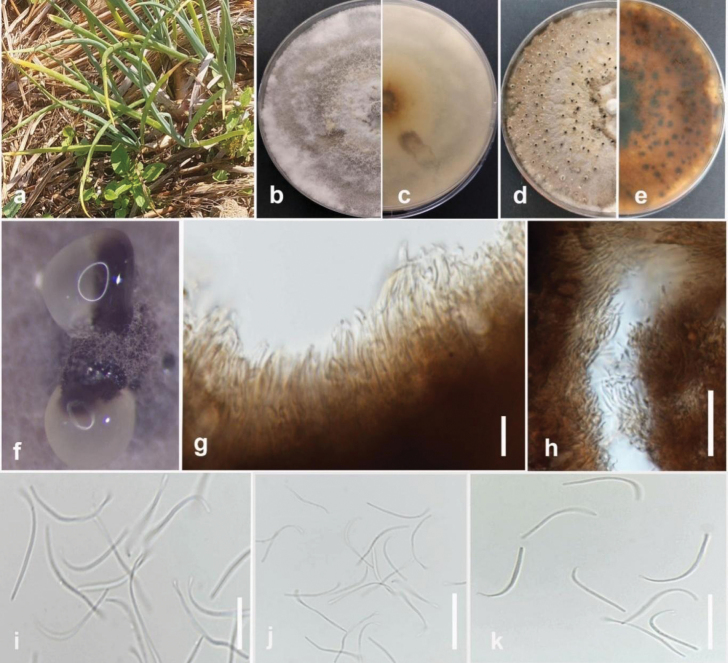
*Diaporthefistulosi* (MFLUCC 24-0244, holotype) **a** wilting symptom in spring onion **b** front, and **c** back view of the colony on PDA after 10 days **d** front and **e** back view of the colony on PDA after 60 days **f** conidiomata **g, h** section view of conidiomata, conidiogenous cells, and paraphyses **i–k** beta conidia. Scale bars: 20 μm.

**Figure 14.**
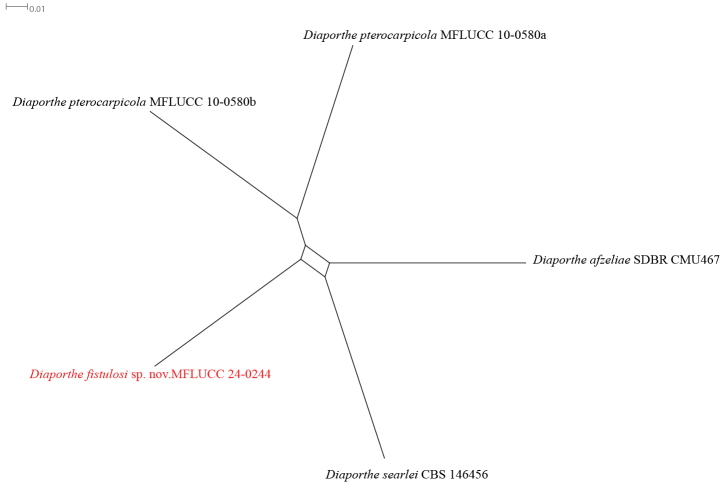
Pairwise homoplasy index (PHI) test of *Diaporthefistulosi* (MFLUCC 24-0244, holotype) and closely related species using both LogDet transformation and splits decomposition. PHI test results (Φw) < 0.05 indicate significant recombination among the species (Φw = 1).

### ﻿ Diaporthe hongkongensis

Taxon classificationFungiSordariomycetesDiaporthaceae

R.R. Gomes, Glienke & Crous, Persoonia 31: 23 (2013)

64DC42E2-1109-5308-B9A3-7E7EBAB5E21B

Index Fungorum: IF802934

Fig. 15

#### Description.

Pathogenic to rambutan (*Nepheliumlappaceum*) and causes fruit rot. Sexual morph not observed. Conidiomata pycnidial, superficial to embedded, solitary to aggregated, pyriform or globose with central ostiole, and cream conidial mass, up to 250 μm in diameter. Conidiophores, hyaline, smooth, septate, subcylindrical. Paraphyses intermingled among conidiophores, hyaline, smooth, branched, septate, with clavate terminal cell, 30–50 × 0.5–1.3 μm (38 × 1 μm, n = 20). Alpha conidia aseptate, hyaline, smooth, ovate to ellipsoidal, granular to guttate, 5.7–7.8 × 1.5–2.5 μm (6.5 × 2 μm, n = 30). Beta conidia filiform, curved at one tip, hyaline, aseptate, rounded at tips, 15–25 × 1–2 μm (20 × 1.5 μm, n = 30). Gamma conidia absent.

**Figure 15.**
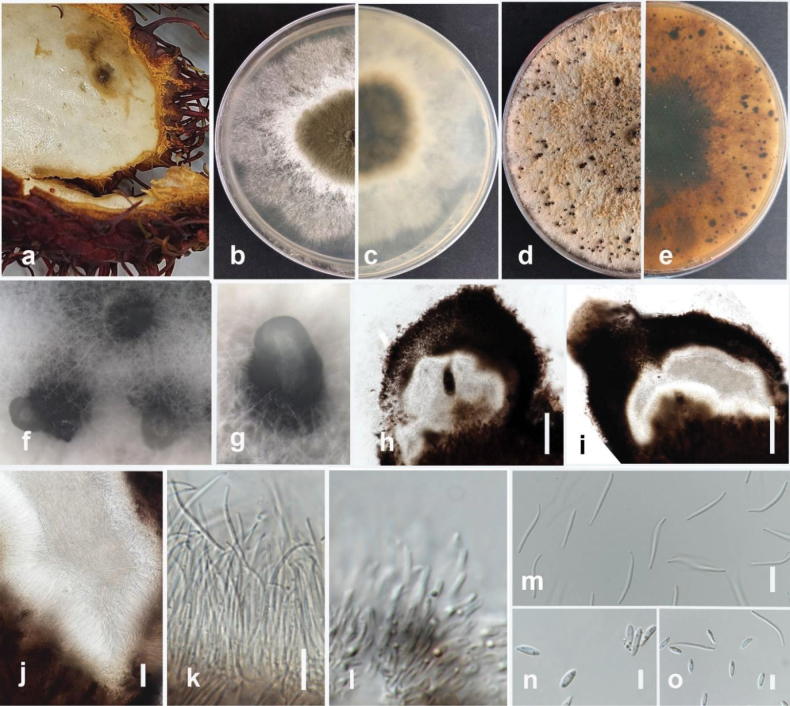
*Diaporthehongkongensis* (MFLUCC 24-0246) **a** fruit brown spot on rambutan **b** front, and **c** back view of the colony on PDA after seven days **d** front and **e** back view of the colony on PDA after 60 days **f, g** conidiomata **h–I** section view of conidiomata **j–l** conidiophores and paraphyses **m** beta conidia **n** alpha conidia **o** alpha and beta conidia. Scale bars: 100 μm **(h, i)**; 20 μm **(j–l)**; 10 μm **(m)**; 5 μm **(n, o)**.

#### Culture characteristics.

Colonies on PDA reach 70–80 mm in diameter after seven days of growth at 25 °C in the dark, felted, fluffy margin, pale olivaceous-grey, with an obvious pale brown concentric ring of dense hyphae, and turn into pale brown with age.

#### Material examined.

Thailand • Chiang Rai Province, Mueang Chiang Rai District, Ban Du, lesion of rambutan (*Nepheliumlappaceum*) fruit, June 2023, Maryam Fallahi, dried culture L2-3 (MFLU 24-0247), living culture, MFLUCC 24-0246.

#### Notes.

Based on phylogenetic analysis, strain MFLUCC 24-0246 grouped in the *Diaporthearecae* species complex with *Diaporthehongkongensis* (CBS 115448, ex-type) with 72% ML bootstrap support and 0.78 BYPP (Fig. 12). The base pair differences between *D.hongkongensis* strains MFLUCC 24-0246 and CBS 115448 revealed a 0.75% (4/533 bp) difference in ITS, a 0.66% (2/301 bp) difference in *tef1*, a 3.84% (16/416 bp) difference in *tub2*, and a 0.46% (2/433 bp) difference in *cal*, and no difference in *his3*. *Diaporthehongkongensis* (MFLUCC 24-0246) and the ex-type strain of *D.hongkongensis* (CBS 115448) are similar. Adding more strains of *D.hongkongensis* in phylogenetic analyses in this study revealed that strains of this species were dispersed throughout the subclade. The relationships among these species remain uncertain, necessitating further analysis to resolve the confusion surrounding their classification and clarify their taxonomic relationships. Recently, a combined gene phylogeny analysis by Dissanayake et al. (2024) redefined the species boundaries of *D.hongkongensis*. The study proposed that eight previously distinct species, *D.australiana*, *D.eucalyptorum*, *D.eucommiae*, *D.lagerstroemiae*, *D.lithocarpus*, *D.rhodomyrti*, and *D.salinicola*, are a single species, *D.hongkongensis* (Dissanayake et al. 2024). *Diaporthehongkongensis* was first isolated from the fruit of *Dichroafebrifuga* in Hong Kong, China (Gomes et al. 2013), which usually causes trunk diseases. It was reported as a cause of top blight of *Cunninghamialanceolata* (Liao et al. 2023), fruit rot in *Prunuspersica* (Zhang et al. 2021), and shoot canker in *Pyruscommunis* (Guo et al. 2020). This study provides a new host and geographical record for *D.hongkongensis* on rambutan in northern Thailand.

### ﻿ Diaporthe rosae

Taxon classificationFungiSordariomycetesDiaporthaceae

Samarak. & K.D. Hyde, Fungal Diversity: 185 (2018)

06D9BCEB-235B-533B-81DA-651C54B11D80

Index Fungorum: IF554072

Facesoffungi Number: FoF13142

Fig. 17

#### Description.

Pathogenic to makhuea kheun (*Solanumxanthocarpum*) and causes dark brown to black stem lesions, circular to irregular necrotic leaf spots with dark margins, and water-soaked fruit lesions that enlarge over time. Sexual morph not observed. Conidiomata pycnidial, multiloculate, scattered, globose, or asymmetrical, black. Peridium consists of brown cells with angular texture on the surface. Conidiophores hyaline, smooth-walled, two-septate, branched, compactly aggregated, cylindrical, straight to sinuous, occasionally reduced to conidiogenous cells. Conidiogenous cells phialidic, subcylindrical, or ampulliform, slightly tapering towards the apex. Beta conidia aseptate, hyaline, smooth-walled, 13–23 × 0.8–1.4 μm (mean = 19 × 1.3 μm, n = 30). Gamma and alpha conidia are absent.

#### Culture characteristics.

Colonies on PDA reach 35–40 mm in diameter after 7 days of growth at 25 °C in the dark, felted, white clots of mycelium arranged outward, becoming pale yellow with age. The reverse is whitish and ozonate.

#### Material examined.

Thailand • Chiang Rai Province, Phan District, Sai Khao, on the stem of makhuea kheun (*Solanumxanthocarpum*). February 2023, Maryam Fallahi, dried culture MF101-1 (MFLU 24-0245), living culture, MFLUCC 24-0243.

#### Notes.

**Figure 16.**
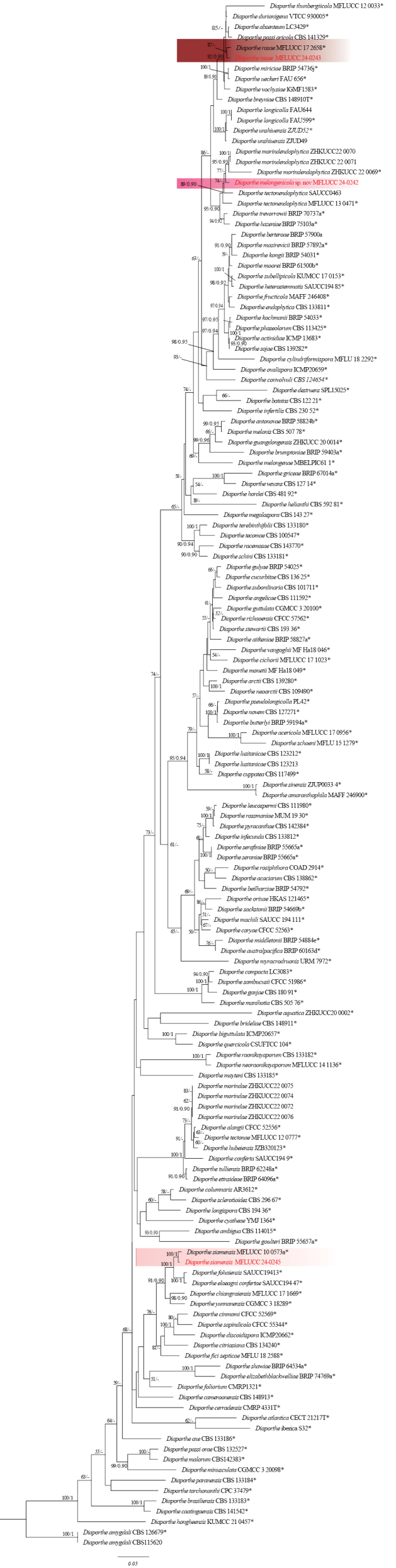
Phylogenetic tree of the *Diaporthesojae* species complex generated by maximum likelihood of combined ITS, *tef1*, *tub2*, *cal*, and *his3* sequence data. The ultrafast maximum likelihood (ML) bootstrap support values ≥50% (BT) and Bayesian posterior probabilities ≥0.90 (BYPP) are shown, respectively, near the nodes. The ex-type strains are marked with an asterisk. The tree is rooted in *D.amygdali* (CBS 126679) and *D.amygdali* (CBS 115620).

Based on phylogenetic analysis of combined ITS, *tef1*, *tub2*, *cal*, and *his3* sequence data, the strain MFLUCC 24-0243 clustered with the ex-type strain of *Diaportherosae* (MFLUCC 17-2658) by 87% ML bootstrap support (Fig. 16). The base pair differences between *D.rosae* strains MFLUCC 24-0243 and MFLUCC 17-2658 revealed a 2.07% (10/481 bp) difference in *cal*, and no difference in ITS and *tub2*. The sequence data for *tef1* and *his3* are not available for the ex-type strain of *D.rosae* (MFLUCC 17-2658). *Diaporherosae* (MFLUCC 24-0243) and the ex-type of *D.rosae* (MFLU 17-2658) are similar. However, *D.rosae* (MFLUCC 24-0243) produced slightly larger beta conidia with an L/W ratio = 14.6 (19 × 1.3 μm in *D.rosae* (MFLUCC 24-0243) vs. 17.5 × 1 μm in *D.rosae* (MFLUCC 17-2658)). *Diaportherosae* was erected by Wanasinghe et al. (2018) as a saprobic taxon that was isolated from a dead pedicel of *Rosa* sp. in Chiang Rai Province, Thailand. Based on the phylogenetic tree of Norphanphoun et al. (2022), *D.rosae* clustered in the *D.sojae* species complex. This study reports *D.rosae* (MFLUCC 24-0243) from makhuea kheun (*Solanumxanthocarpum*), identifying this plant as a new host.

**Figure 17.**
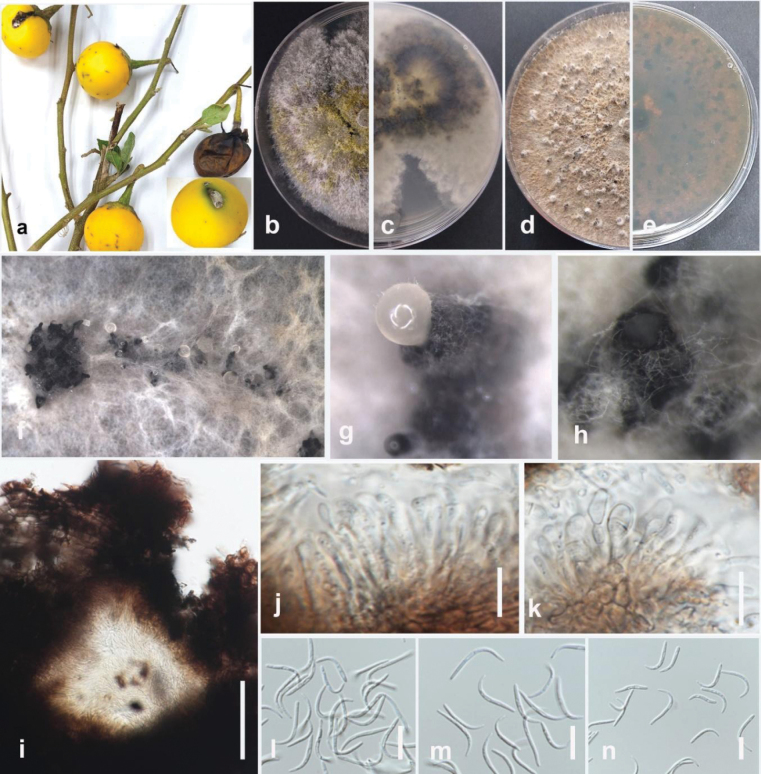
*Diaportherosae* (MFLUCC 24-0243) **a** stem lesion on makhuea kheun **b** front, and **c** back view of the colony on PDA after 10 days **d** front and **e** back view of the colony on PDA after 60 days **f–h** conidiomata **I** section view of conidiomata **j, k** conidiogenous cells **m** beta conidia. Scale bars: 50 μm (**i**); 10 μm (**j–n**).

### ﻿ Diaporthe siamensis

Taxon classificationFungiSordariomycetesDiaporthaceae

Udayanga, Xing Z. Liu & K.D. Hyde, Cryptog. Mycol. 33(3): 298 (2012)

8937D984-430A-5361-9EB6-7DE39EF5AE1D

Index Fungorum: IF800826

Facesoffungi Number: FoF02398

Fig. 18

#### Description.

Pathogenic to rambutan (*Nepheliumlappaceum*) and causes fruit rot. Sexual morph not observed. Conidiomata pycnidial, subglobose, flasky, or erratically shaped, with individual or multiple cavities. Conidiophores cylindrical, hyaline, simple, in dense aggregates, 1.5–1.8 μm. Conidiogenus cells hyaline, phialidic, cylindrical. Paraphyses hyaline, sub-cylindrical, septate, reaching above conidiophores, straight, flexuous, branched, up to 33 μm in length. Beta conidia aseptate, hyaline, hamate, or curved, with an acutely rounded apex and truncated base, 18–32 × 1–1.8 µm (mean = 25.5 × 1.3 μm, n = 30). Gamma and alpha conidia not observed.

**Figure 18.**
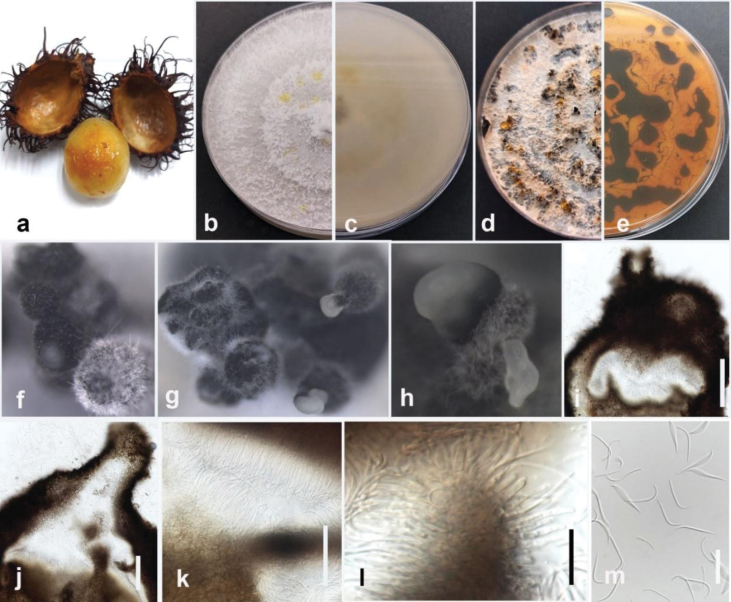
*Diaporthesiamensis* (MFLUCC 24-0245) **a** fruit rot in rambutan **b** front, and **c** back view of the colony on PDA after 10 days **d** front and **e** back view of the colony on PDA after 60 days **f–h** conidiomata **i, j** section view of conidiomata **k, l** conidiophores and paraphyses **m** beta conidia. Scale bars: 100 μm **(i, j)**; 50 μm **(k)**; 20 μm **(l, m)**.

#### Culture characteristics.

Colonies on PDA reach 60–65 mm in diameter after 7 days of growth at 25 °C in the dark, cottony, white to cream, with lobate margins. The reverse is greenish yellow, with emerging dark pigmentation spots, along with the production of enormous black stromata on PDA.

#### Material examined.

Thailand • Chiang Rai Province, Mueang Chiang Rai District, Ban Du, Fruit rot on rambutan (*Nepheliumlappaceum*), June 2023, Maryam Fallahi, dried culture L1-2 (MFLU 24-0246), living culture, MFLUCC 24-0245.

#### Notes.

MFLUCC 24-0245 strain clustered with *Diaporthesiamensis* (MFLUCC 10-0573a, ex-type) with 100% ML bootstrap support, and 1.0 BYPP (Fig. 16). The base pair differences between *D.siamensis* strains MFLUCC 24-0245 and MFLUCC 10-0573a revealed a 0.38% (2/519 bp) difference in ITS, a 1.7% (4/234 bp) difference in *tef1*, a 0.64% (3/469 bp) difference in *tub2*, and no difference in *cal*. The sequence data of *his3* is not available for *D.siamensis* (MFLUCC 10-0573a). *D.siamensis* (MFLUCC 24-0245) has larger Beta conidia with an L/W = 19.6 (18–32 × 1–1.8 µm in *D.siamensis* (MFLUCC 24-0245) vs. 15–18 × (1.5–) 2 μm in *D.siamensis* (MFLU 12–0121, holotype)), and it did not produce gamma and alpha conidia. Based on the phylogenetic tree of Norphanphoun et al. (2022), *D.siamensis* grouped in the *D.sojae* species complex. Previous reports indicated that *D.siamensis* exhibited the ability to cause disease in *Citrussinensis* (Cui et al. 2021) and *Dasymaschalon* sp. (Udayanga et al. 2012). Additionally, it was identified as an endophyte in *Pandanus* sp. in Thailand (Tibpromma et al. 2018) and *Garciniaparvifolia* in Malaysia (Udayanga et al. 2012). *Diaporthesiamensis* from rambutan was isolated in Thailand by Abeywickrama et al. (2023).

### ﻿ Diaporthe melongenicola

Taxon classificationFungiSordariomycetesDiaporthaceae

Fallahi, Jayawar. & K.D. Hyde sp. nov.

47BACC89-2A23-5C41-A9D0-F6062A9BBFF2

Index Fungorum: IF903266

Fig. 20

#### Etymology.

‘*melongenicola*’ refers to the host plant species from which the fungus was isolated.

#### Description.

Pathogenic to makhuea (*Solanummelongena*) and causes blight, stem cankers, and fruit rot. Sexual morph not observed. Conidiomata pycnidial, superficial to embedded, solitary to aggregated, with single or multiple cavities, globose, with cream conidial mass, up to 200 μm in diameter. Conidiophores hyaline, smooth, straight, unbranched, rounded at the tip, and wider at the base, septate, 9–20 × 1.2–2.9 μm (mean = 13.4 × 1.9 μm, n = 15). Paraphyses intermingled among conidiophores, hyaline, smooth, septate, 9–33 × 1.4–1.5 μm (mean = 24 × 1.16 μm, n = 15). Beta conidia filiform, curved at one tip, hyaline, aseptate, rounded at tips, 15–27 × 1–2 μm (mean = 20 × 1.5 μm, n = 30). Alpha and gamma conidia are absent.

#### Culture characteristics.

Colonies on PDA reach 70–80 mm in diameter after seven days of growth at 25 °C in the dark, fluffy and slightly felted with age, circular in shape, with cottony growth of the aerial mycelium in rings, grey to olivaceous grey, pale brown with age. The reverse is reddish brown, with many black dots.

#### Material examined.

Thailand • Chiang Rai, Mueang Chiang Rai District, Doi Hang, stem canker in makhuea (*Solanummelongena*), January 2023, Maryam Fallahi, dried culture MF90-2 (MFLU 24-0244, holotype), ex-holotype culture, MFLUCC 24-0242.

#### Notes.

**Figure 19.**
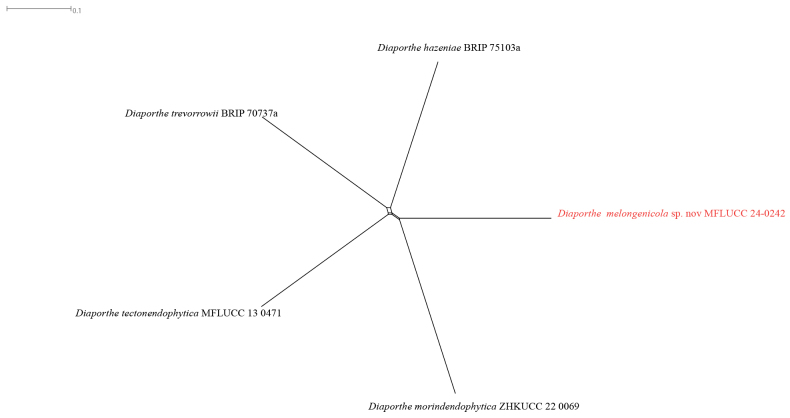
Pairwise homoplasy index (PHI) test of *Diaporthemelongenicola*MFLUCC 24-0242 and closely related species using both LogDet transformation and splits decomposition. PHI test results (Φw) < 0.05 indicate significant recombination among the species (p = 1).

In the phylogenetic tree generated in this study, strain MFLUCC 24-0242 formed a distinct branch within the *D.sojae* species complex in the subclade that includes the type strains of *D.morindendophytica* (ZHKUCC 22-0069, holotype) and *D.tectonendophytica* (MFLUCC 13-0471, ex-type) with 89% ML bootstrap support and 0.90 BYPP (Fig. 16) and is introduced here as a new species, namely *Diaporthemelongenicola*. The base pair differences between *Diaporthemelongenicola* (MFLUCC 24-0242) with type strains of closely related species are presented in Table 4. A pairwise homoplasy index (PHI) test indicated no significant recombination (Φw = 1.0) between *D.melongenicola* (MFLUCC 24-0242, holotype) and its closely related taxa (Fig. 19). These species are different from our isolate by producing alpha conidia and diverse culture characteristics. Considering both morphology and molecular data, *D.melongenicola* (MFLUCC 24-0242) could be identified as a novel species on makhuea (*Solanummelongena*) in northern Thailand.

**Table 4.** Pairwise differences in the DNA sequence data of *Diaporthemelongenicola* (MFLUCC 24-0242) with type strains of closely related species.

	*D.morindendophytica* (ZHKUCC-22-0069)	*D.tectonendophytica* (MFLUCC-13-0471)
**ITS**	0.99%	1.87%
***tef1***	16%	0%
***tub2***	1.5%	2.77%
***cal***	0%	1.26%
***his3***	1.2%	0%

**Figure 20.**
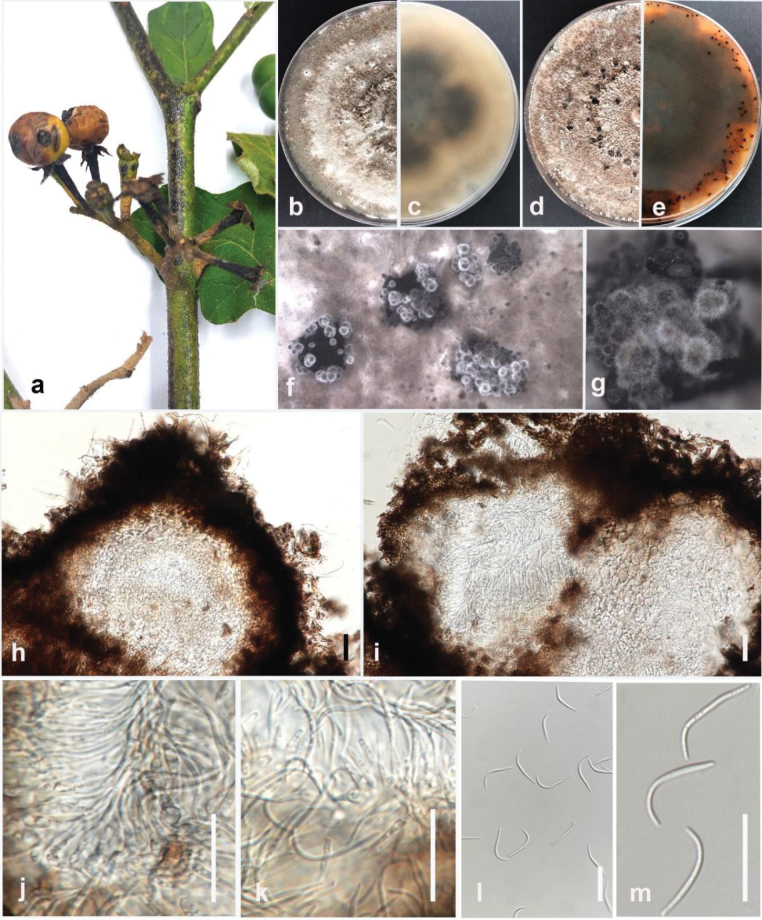
*Diaporthemelongenicola* (MFLUCC 24-0242, holotype) **a** blight and stem canker in makhuea **b** front, and **c** back view of the colony on PDA after 10 days **d** front and **e** back view of the colony on PDA after 60 days **f–h** conidiomata **i, j** section view of conidiomata **k, l** conidiophores and paraphyses **m** beta conidia. Scale bars: 20 μm (**i, j**); 10 μm (**k–m**).

### ﻿ Fusarium

Taxon classificationFungiSordariomycetesNectriaceae

Link, Mag. Gesell. naturf. Freunde, Berlin 3(1–2): 10 (1809)

4CA45B88-9C47-521A-8B3F-4D3D659F3CF6

#### Notes.

In the present study, five species of *Fusarium*, including *Fusariumbubalinum*, *F.languescens*, *F.nirenbergiae*, *F.sulawesiense*, and *F.tanahbumbuense*, are reported from different hosts in Thailand.

### ﻿ Fusarium bubalinum

Taxon classificationFungiSordariomycetesNectriaceae

J.W. Xia, L. Lombard, Sand.-Den., X.G. Zhang & Crous, Persoonia 43: 195 (2019)

62018EAD-EB99-58C7-B741-C968F7943B66

Index Fungorum: IF831831

Fig. 22

#### Description.

Pathogenic to dragon fruit (*Hylocereustrigonus*) and causes stem rot. Sexual morph not observed. Conidiophores on aerial mycelium unbranched, sympodial, or irregularly branched, comprising terminal or lateral phialides that are frequently reduced to single phialides. Conidiogenous cells mono- or polyphialidic, subulate to subcylindrical, smooth and thin-walled, 5–25 × 1.5–3.5 μm. Aerial conidia ellipsoidal to falcate, slender, curved dorsoventrally, tap towards both ends, blunt to conical and straight to slightly curved apical cell, with a blunt to papillate basal cell, 0–7 septate, 8–28.5 × 1.3–2.8 µm (mean = 16 × 2 μm, n = 30). Microcyclic conidiogenesis often occurs. Sporodochia and chlamydospores are absent.

#### Culture characteristics.

Colonies on PDA reach 80 mm in diameter after 7 days of growth at 25 °C in the dark, cottony, white to buff, floccose, and radiate with moderate aerial mycelium, filiform, and margins irregular, having sparse aerial mycelium and high sporulation on the surface of SNA medium. The reverse is a pale primrose.

#### Material examined.

Thailand • Chiang Rai, Mueang Chiang Rai District, Doi Hang. On stem rot in dragon fruit, February 2023, Maryam Fallahi, dried culture MF35-5 (MFLU 24-0249), living culture MFLUCC 24-0230.

#### Notes.

**Figure 21.**
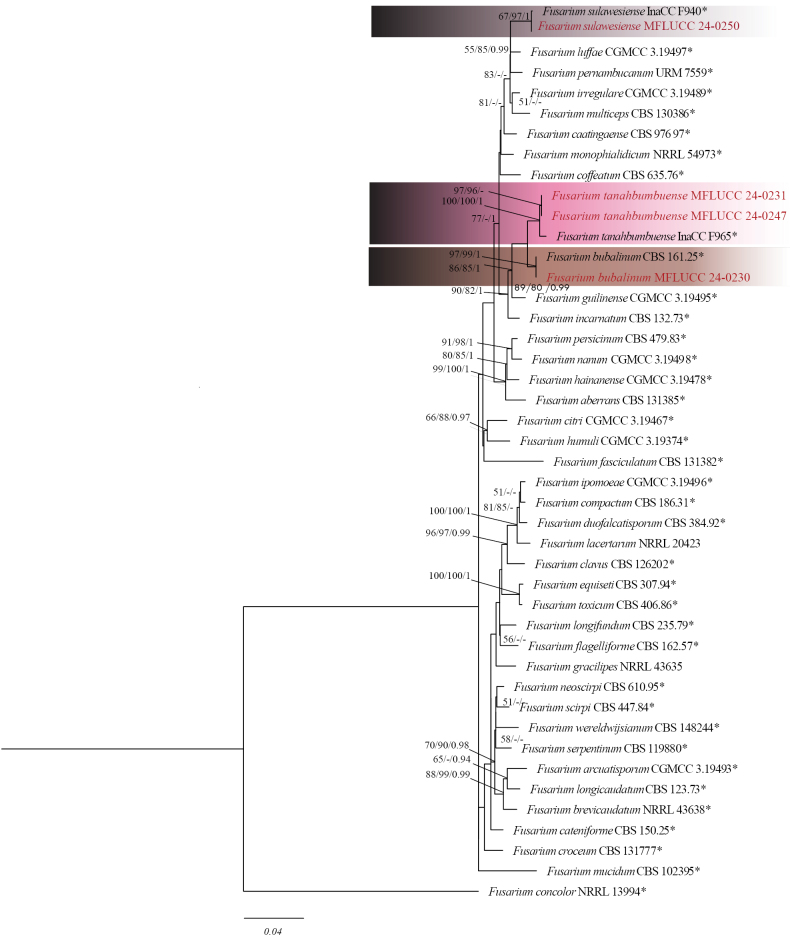
Phylogenetic tree of the *Fusariumincarnatum* species complex generated by maximum likelihood of combined *tef1*, *rpb1*, and *rpb2* sequence data. The ultrafast maximum likelihood (ML) and IQ bootstrap support values ≥50% (BT), as well as Bayesian posterior probabilities ≥ 0.95 (BYPP) are shown, respectively, near the nodes. The ex-type strains are marked with an asterisk. The tree is rooted in *F.concolor* (NRRL 13994).

Based on phylogenetic analysis, strain MFLUCC 24-0230 clustered in the same subclade with *Fusariumbubalinum* (CBS 161-25, ex-type) in *Fusariumincarnatum* species complex with 97% ML, 99% IQ bootstrap support and 0.99 BYPP (Fig. 21). The base pair differences between *F.bubalinum* strains MFLUCC 24-0230 and ex-type CBS 161-25 showed that they are identical in *tef1* and *rpb2*, and sequence data of *rpb1* are not available for *F.bubalinum* (CBS 161-25, ex-type). *Fusariumbubalinum* (MFLUCC 24-0230) is similar to the ex-type strain of *F.bubalinum* in morphology (CBS 161-25). *Fusariumbubalinum* was introduced as a new species in the *Fusariumincarnatum-equiseti* complex, and the type strain was isolated from an unknown substrate in Australia (Xia et al. 2019). However, details regarding its host are scarce. Recently, it was reported in association with sheath rot disease of rice in Indonesia (Pramunadipta et al. 2022). In this study, we report *F.bubalinum* causing stem rot in dragon fruit in northern Thailand as a new host and geographical record.

**Figure 22.**
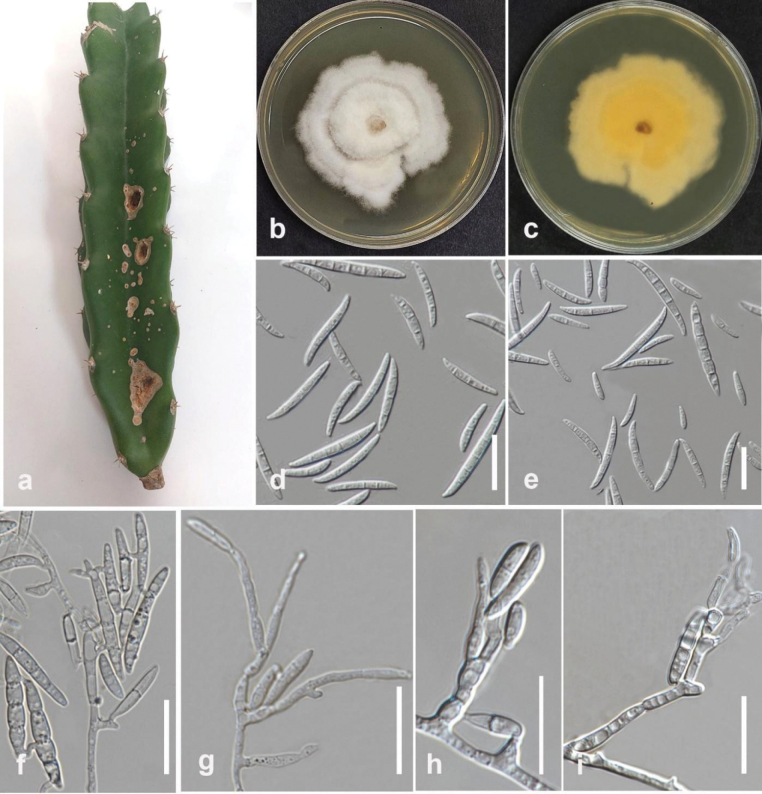
*Fusariumbubalinum* (MFLUCC 24-0230) **a** stem rot in dragon fruit **b** upper, and **c** reverse views of the colony after seven days of growth on PDA at 25 °C **d, e** conidia **f** conidiophores and conidia anastomose and germination **g–i** conidiophores, conidiogenous cells, and aerial conidia. Scale bars: 20 μm.

### ﻿ Fusarium sulawesiense

Taxon classificationFungiSordariomycetesNectriaceae

Sand.-Den., L. Lombard, Kema & Crous [as ‘ sulawense’], Persoonia 43: 65 (2019)

9F4717E2-2E48-563F-B562-B9ED0E9E65B4

Index Fungorum: IF830777

Facesoffungi Number: FoF13412

Fig. 23

#### Description.

Pathogenic to Mangosteen (*Garciniamangostana*) and causes small, water-soaked lesions on the fruit surface; lesions may appear slightly sunken and surrounded by a yellow or brown halo. Sexual morph not observed. Conidiophores on aerial mycelium plentiful, septate, verticillately or irregularly branched. Conidiogenous cells mono- or polyphialidic, sub-cylindrical, smooth, thin-walled, 8–20 × 1.5–4 µm (mean = 14 × 3 μm, n = 20). Conidia on aerial mycelium falcate or fusiform, 1–5 septate: 1-septate conidia 8.5–20 × 2.2–4.7 µm (mean = 13.5 × 3.5 μm, n = 30), 5-septate conidia 32–42 × 2.5–4.5 µm (mean = 35 × 4.1 μm, n = 30). Sporodochia are formed on CLA after 7 days and have a pale orange color. Macroconidia in sporodochia falcate, with apical cells gently curved, papillate, and basal cells slightly curved, foot-shaped, 3–7 septate, 30–40.5 × 2.5–4 µm (mean = 33 × 3.6 μm, n = 30). Chlamydospores are absent.

**Figure 23.**
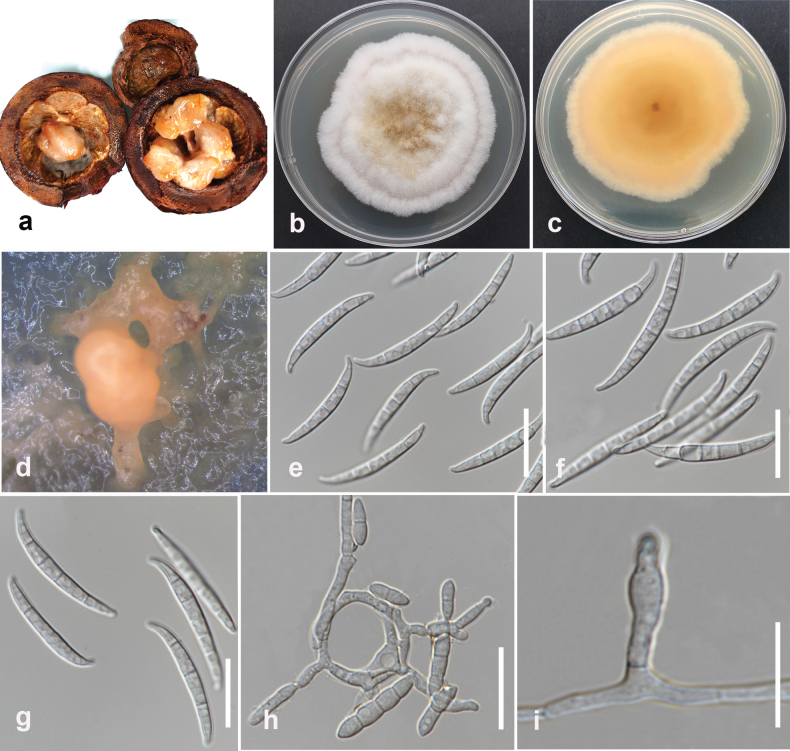
*Fusariumsulawesiense* (MFLUCC 24-0250) **a** fruit rot in Mangosteen **b** front and **c** back views of the colony after 7 days of growth on PDA at 25 °C **d** spordochium **e–g** sporodochial conidia **h, i** Conidiogenous cells and conidia. Scale bars: 20 μm (**e–h**); 10 μm (**i**).

#### Culture characteristics.

Colonies on PDA reach 65 mm in diameter after 7 days of growth at 25 °C in the dark, cottony, white to pale pink, yellow, or pale brown in the center, and with a radial orange color on the reverse.

#### Material examined.

Thailand • Chiang Rai, Mueang Chiang Rai District, Ban Du, on fruit rot of Mangosteen (*Garciniamangostana*), June 2023, Maryam Fallahi, dried culture L5-6 (MFLU 24-0253), living culture, MFLUCC 24-0250.

#### Notes.

Based on phylogenetic analysis, MFLUCC 24-0250 clustered with *Fusariumsulawesiense* (InaCC F940, holotype) in *F.incarnatum* species complex with 67% ML, 97% IQ bootstrap support, and 0.99 BYPP (Fig. 21). The base pair differences between *F.sulawesiense* strains MFLUCC 24-0250 and the ex-type InaCC F940 revealed a 0.36% (2/554 bp) difference in *tef1* and no difference in *rpb2*. The sequence of the *rpb1* gene is not available for the type strain. *Fusariumsulawesiense* (MFLUCC 24-0250) is similar to the ex-type strain (InaCC F964). However, the ex-type strain did not describe septate conidia (Maryani et al. 2019b). *Fusariumsulawesiense* was first reported from bananas in Indonesia (Maryani et al. 2019b). In Thailand, it was isolated from pineapple (Abeywickrama et al. 2023), and this study provides the first host association of *F.sulawesiense* with Mangosteen (*Garciniamangostana*).

### ﻿ Fusarium tanahbumbuense

Taxon classificationFungiSordariomycetesNectriaceae

Sand.-Den., L. Lombard, Kema & Crous, Persoonia 43: 63 (2019)

07B14C6A-A0F5-59FC-A272-1641F6F7E7F7

Index Fungorum: IF828962

Fig. 24

#### Description.

Pathogenic to durian (*Duriozibethinus*) and pepper (*Capsicumannuum*), and causes small, water-soaked lesions on leaves and stems that enlarge into necrotic spots with dark margins, potentially leading to defoliation and stem girdling under favorable, humid conditions. Sexual morph not observed. Conidiophores on aerial mycelium septate, irregular, verticillately branched. Conidiogenous cells mono- or polyphialidic, subulate or subcylindrical, smooth and thin-walled, 7–24 × 2–4 µm (mean = 13 × 3 µm, n = 15). Conidia on aerial mycelium, ellipsoidal to falcate, smooth and thin-walled, 1–3 septate, 7–27 × 2–4 µm (mean = 33 × 3 µm, n = 30). Sporodochia are formed plentifully on CLA after 7 days and have a pale orange color. Conidia on sporodochia falcate, produced by both mono- and polyphialides, apical cells conical to papillate, basal cells indistinct or foot-shaped, 3–5-septate, 26–38 × 2–3.7 µm (mean = 33 × 3 µm, n = 30). Chlamydospores are absent.

**Figure 24.**
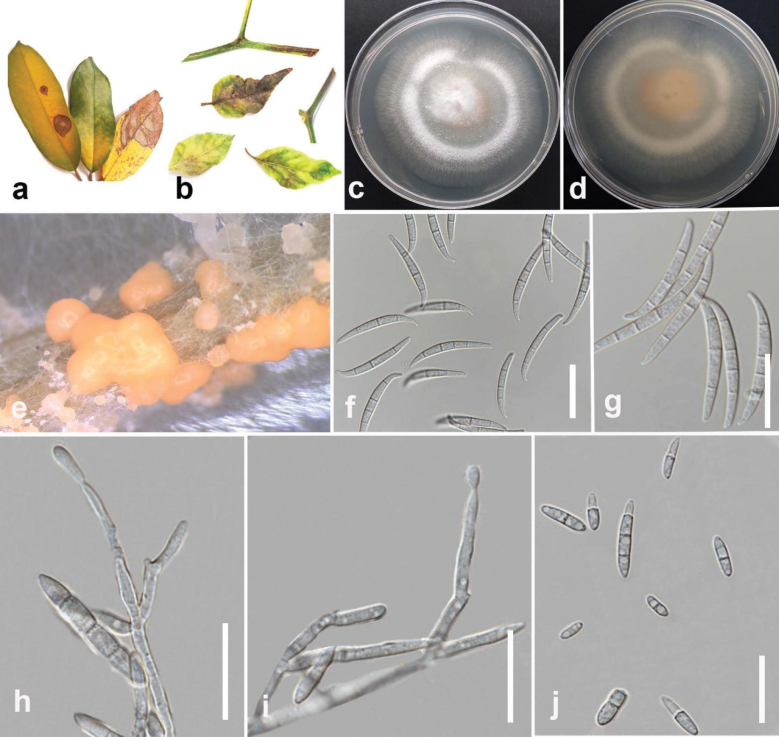
*Fusariumtanahbumbuense* (MFLUCC 24-0231) **a** leaf spots on durian **b** stem and leaf spots on pepper **c** front, and **d** back views of the colony after 10 days of growth on PDA at 25 °C **e** spordochium **f–g** spordochium conidia **h, i** conidiophores and conidiogenous cells. **j** aerial conidia. Scale bars: 20 μm.

#### Culture characteristics.

Colonies on PDA reach 50 mm in diameter after 7 days of growth at 25 °C in the dark, cottony and rosy buff in the center, becoming white towards the margin, with moderate aerial mycelium, and appearing wet with age. The reverse is rosy buff, becoming white towards the margins.

#### Material examined.

Thailand • Chiang Rai, Mueang Chiang Rai District, Doi Hang, on leaf spot on durian (*Duriozibethinus*), December 2022, Maryam Fallahi, dried culture MF31-1 (MFLU 24-0248), living culture MFLUCC 24-0231 • Amphoe Mueang Sakon Nakhon, Chang Wat Sakon Nakhon, leaf spot on pepper (*Capsicumannuum*), February 2023, Maryam Fallahi, dried culture MF145-1 (MFLU 24-0250), living culture MFLUCC 24-0247.

#### Notes.

In the present study, strains MFLUCC 24-0231 and MFLUCC 24-0247 clustered with *Fusariumtanahbumbuense* (InaCC: F965, ex-type) in *F.incarnatum* complex with 100% ML, 100% IQ bootstrap support, and 1.0 BYPP (Fig. 21). The base pair differences between *F.tanahbumbuense* strains MFLUCC 24-0231 and ex-type InaCC: F965 revealed a 0.36% (2/541 bp) difference in *tef1*, a 0.71% (6/838 bp) difference in *rpb2*, and a 0.27% (2/742 bp) difference in *rpb1*. The base pair differences between *F.tanahbumbuense* (MFLUCC 24-0247) and *F.tanahbumbuense* (InaCC: F965, ex-type) revealed a 0.42% (2/470 bp) difference in *tef1*, a 0.62% (8/801 bp) difference in *rpb2*, and a 0.27% (2/742 bp) difference in *rpb1. Fusariumtanahbumbuense* was first reported from Indonesia on an infected pseudostem of *Musa* sp. (Maryani et al. 2019a). This study provides two new hosts and geographical records for *F.tanahbumbuense* on durian and pepper.

### ﻿ Fusarium languescens

Taxon classificationFungiSordariomycetesNectriaceae

L. Lombard & Crous, Persoonia 41: 28 (2018)

FCFAA968-04FC-544F-A40A-E4E47577AB72

Index Fungorum: IF826843

Fig. 26

#### Description.

Associated with tuber rot of lesser yam (*Dioscoreaesculenta*). Sexual morph not observed. Conidiophores on aerial mycelium unbranched or slightly branched, comprise terminal or intercalar monophialides, frequently reduced to single phialides. Aerial phialides subulate to subcylindrical, smooth, and thin-walled, 6.5–18 × 2–3.8 µm (Mean = 15 × 2.5 µm, n = 15), with unnoticeable or absent periclinal thickening. Microconidia ellipsoidal to falcate, hyaline, smooth, thin-walled, 0-septate, 3.6–9 × 2–3.4 µm (Mean = 6 × 2.5 µm, n = 30), formed in a small false head on the tips of the phialides on SNA. Sporodochia light orange on carnation leaves. Conidiophores in sporodochia verticillately branched, comprising a short, smooth, and thin-walled stipe, carrying apical whorls of 2–3 monophialides or scarcely single lateral monophialides. Sporodochial phialides subulate to subcylindrical, smooth, and thin-walled. Sporodochial conidia falcate, curved dorsiventrally, with almost parallel sides tapering a little towards both ends, with a blunt papillate and curved apical cell. Basal cells are blunt to foot-like, 1–5 septate, hyaline, smooth, and thin-walled; 1-septate conidia 18–23 × 3–4 µm (mean = 20 × 3 µm, n = 20); 2-septate conidia 15–22 × 3–4 µm (mean = 18 × 3 µm, n = 20); 3-septate conidia 25–37 × 3–5 µm (mean = 31 × 4 µm, n = 20); 5-septate conidia 33–41 × 4–5 µm (mean = 35 × 5 µm, n = 20). Chlamydospores globose to subglobose, formed terminally, 7–8 µm in diameter.

#### Culture characteristics.

Colonies on PDA reach 80–85 mm in diameter after 7 days of growth at 25 °C in the dark, white to pale vinaceous, floccose with plentiful aerial mycelium. The margins of colonies are irregular, serrate, or filiform. The reverse is pale rosy.

#### Material examined.

Thailand • Chiang Rai Province, Mueang Chiang Rai District, Doi Hang, on lesser yam (*Dioscoreaesculenta*), February 2023, Maryam Fallahi, dried culture MF67-4 (MFLU 24-0252), living culture, MFLUCC 24-0249.

#### Notes.

**Figure 25.**
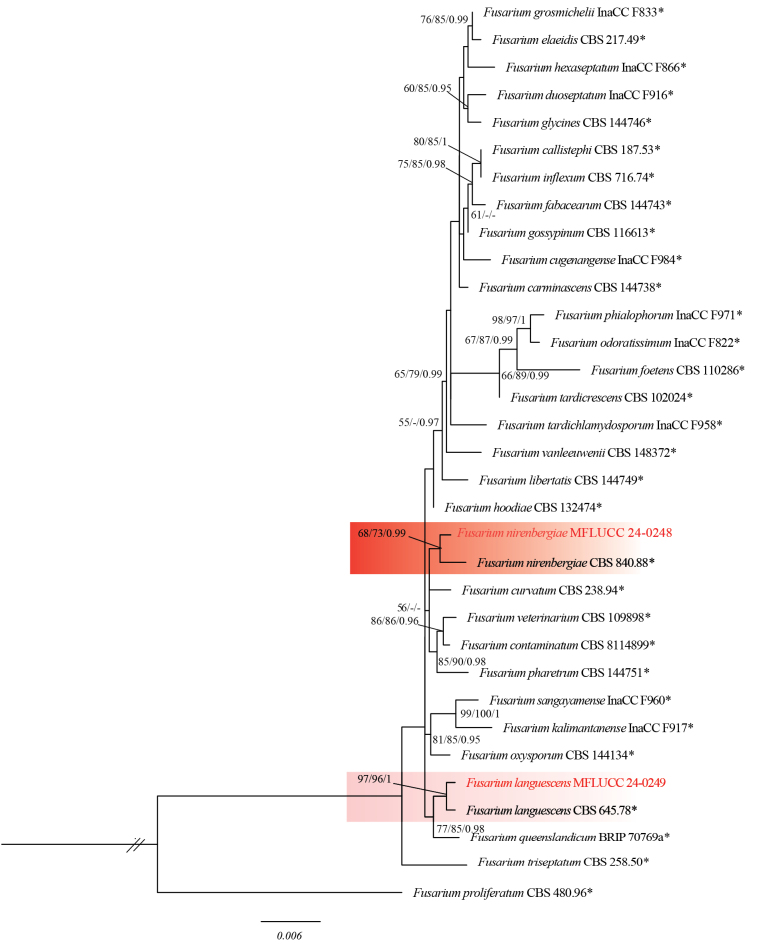
Phylogenetic tree of the *Fusariumoxysporum* species complex generated by maximum likelihood of combined *tef1*, *rpb1*, and *rpb2* sequence data. The ultrafast maximum likelihood (ML) and IQ bootstrap support values ≥ 50% as well as Bayesian posterior probabilities ≥ 0.95 (BYPP) are shown, respectively, near the nodes. The ex-type strains are marked with an asterisk. The tree is rooted in *Fusariumproliferatum* (CBS 480 96).

Based on the phylogenetic tree generated for *Fusariumoxysporum* species complex (FOSC), strain MFLUCC 24-0249 clustered with *F.languescens* (CBS 645.78, ex-type) with 97% ML, 96% IQ bootstrap support, and 1.0 BYPP (Fig. 25). The base pair differences between *F.languescens* strains MFLUCC 24-0249 and ex-type CBS 645.78 revealed a 0.33% (2/601 bp) difference in *tef1*, a 0.25% (2/795 bp) difference in *rpb1*, and no difference in *rpb2*. Phylogenetic analysis by Lombard et al. (2019) revealed that *F.languescens* establishes a subclade as highly supported, which mostly includes strains related to tomato wilt. *Fusariumlanguescens* shows morphological overlap with several species. Hence, phylogenetic inference is required to identify them correctly. Recently, several putative species, six belonging to FOSC (*F.aff.cugenangense*, *F.aff.curvatum*, *F.aff.gossypinum*, *F.aff.nirenbergiae*, *F.aff.odoratissimum*, and *Fusarium* aff. sp.), *F.aff.asiaticum*, *F.aff.commune*, *F.aff.fujikuroi*, *F.aff.solani*, and *F.* aff. Verticillioides, were reported from Chinese yam (*Dioscoreapolystachya* Thunb.) in China (Dongzhen et al. 2020). Dongzhen et al. (2020) stated these species are potentially new taxa, and they used “species affinis” or “aff. sp.” for short to the tentative nature of their species identifications. This is the first report of *F.languescens* on lesser yam in the world and Thailand.

**Figure 26.**
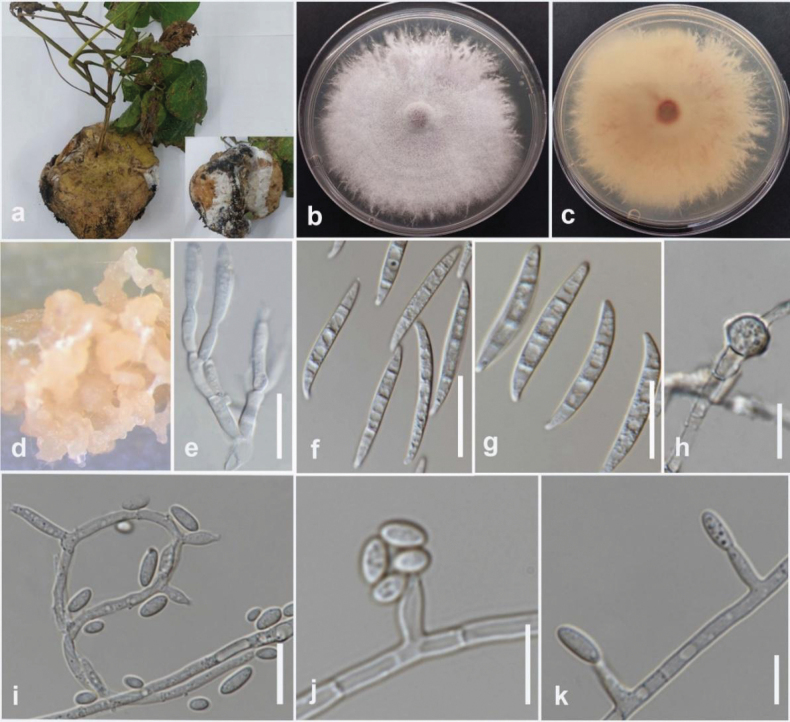
*Fusariumlanguescens* (MFLUCC 24-0249) **a** tuber rot in lesser yam **b** front, and **c** back views of the colony after seven days of growth on PDA at 25 °C **d** sporodochium **e** sporodochial conidiophore and phialides **f, g** sporodochial conidia **h** chlamydospore **i–k** aerial conidiophores, conidiogenous cells, and conidia. Scale bars: 20 μm **(e–g**); 10 μm (**h, i**); 5 μm (**j, k**).

### ﻿ Fusarium nirenbergiae

Taxon classificationFungiSordariomycetesNectriaceae

L. Lombard & Crous, Persoonia 41: 29 (2018)

5D72D9A8-A6A8-5D9A-A9B5-27F53DBC389A

Index Fungorum: IF826845

Fig. 27

#### Description.

Pathogenic to spring onion (*Alliumfistulosum*) and causes yellowing, curling, and wilting of leaves, often accompanied by basal rot and reddish-brown discoloration of the roots and bulb plate. Sexual morph not observed. Conidiophores on aerial mycelium unbranched or slightly branched, comprise terminal or intercalary monophialides, often reduced to single phialides. Aerial phialides, subulate to subcylindrical, smooth, thin-walled, 9–23 × 1.5–2.5 µm, with unnoticeable or absent periclinal thickening. Aerial conidia formed in small false heads on the tips of the phialides, 0–1-septate 0-septate conidia: 5–9 × 2–4 μm (mean = 8 × 3 μm, n = 20), 1-septate conidia: 9–14 × 2–4 μm (mean = 12 × 3 μm, n = 20). Macroconidia falcate, curved dorsoventrally, with apical cell blunt to papillate, curved, basal cell blunt to foot-like, hyaline, smooth, and thin-walled, 3–5-septate: 3-septate conidia: 31–41 × 3.5–4 μm (mean = 35 × 4 μm, n = 20), 4-septate conidia: 35–45 × 3–5 μm (mean = 38 × 4 μm, n = 20), 5-septate conidia: 42–55 × 3–4 μm (mean = 50 × 4 μm, n = 20). Sporodochia and chlamydospores were not observed.

**Figure 27.**
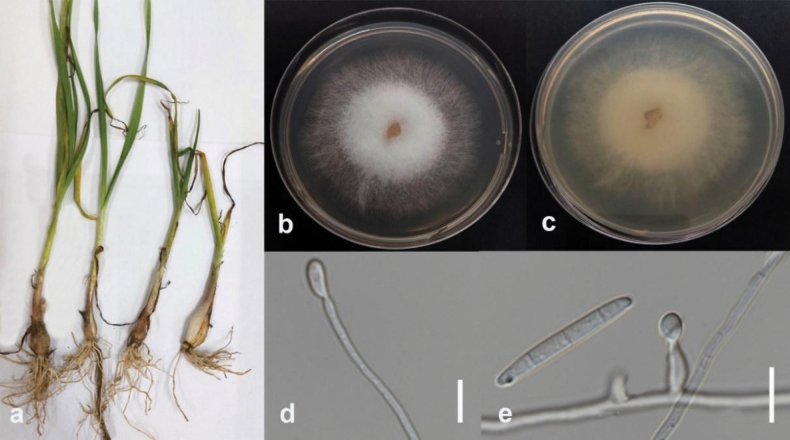
*Fusariumnirenbergiae* (MFLUCC 24-0248) **a** spring onion wilting **b** front and **c** back views of the colony after seven days of growth on PDA at 25 °C **d, e** aerial conidiophores, conidiogenous cells, and conidia. Scale bars: 10 μm.

#### Culture characteristics.

Colonies on PDA reach 65 mm in diameter after 7 days of growth at 25 °C in the dark, white to pale vinaceous, with abundant aerial mycelium and filiform margins. Reverse is pale vinaceous.

#### Material examined.

Thailand • Chiang Rai Province, Mueang Chiang Rai District, Doi Hang, on spring onion (*Alliumfistulosum*), February 2023, Maryam Fallahi, dried culture MF112-2 (MFLU 24-0251), living culture MFLUCC 24-0248.

#### Notes.

In the phylogenetic tree generated for *Fusariumoxysporum* species complex, strain MFLUCC 24-0248 clustered with the ex-type strain of *Fusariumnirenbergiae* (CBS 840 88) with 68% ML, 73% IQ bootstrap support, and 0.99 BYPP (Fig. 25). The base pair differences between *F.nirenbergiae* strains MFLUCC 24-0248 and CBS 840 88 revealed a 0.16% (1/612 bp) difference in *tef1* and a 0.57% (5/876 bp) difference in *rpb2*. The sequence data of *rpb1* is not available for *F.nirenbergiae* (CBS 840 88). *Fusariumnirenbergiae* (MFLUCC 24-0248) did not produce sporodochia or chlamydospores; however, the ex-type strain of *F.nirenbergiae* did produce both structures. (Lombard et al. 2019). Phylogenetically, *F.nirenbergiae* is closely related to *F.curvatum*; however, they are distinguished based on morphology and molecular analysis (Lombard et al. 2019). *Fusariumnirenbergiae* is globally recognized as a causative agent of wilting on various hosts (Lombard et al. 2019; Aiello et al. 2021). To the best of our knowledge, this study represents the first report of Fusarium wilt on spring onion caused by *F.nirenbergiae*.

### ﻿ Neopestalotiopsis

Taxon classificationFungiSordariomycetesPestalotiopsidaceae

Maharachch., K.D. Hyde & Crous, Studies in Mycology 79: 135.

421E718A-E31F-5983-AC8E-B14B0F881D03

#### Notes.

This study reports three new host records for *Neopestalotiopsisformicarum* and *N.zakeelii*, and three unidentified species (*Neopestalotiopsis* sp. 1, *Neopestalotiopsis* sp. 2, and *Neopestalotiopsis* sp. 3) that are in need of further collections’ analyses to be formally described. Herein, one new species, *Neopestalotiopsistheobromicola*, is introduced.

### ﻿ Neopestalotiopsis formicidarum

Taxon classificationFungiSordariomycetesPestalotiopsidaceae

Maharachch., K.D. Hyde & Crous [as ‘formicarum’], Mycol. 79: 140 (2014)

185522EE-940B-57A8-A014-E27D98ABBCD8

Index Fungorum: IF821673

Facesoffungi Number: FoF10804

Fig. 29

#### Description.

Pathogenic to rambutan (*Nepheliumlappaceum*) and associated with dry leaf spots of lemon drop mangosteen (*Garciniaintermedia*). Sexual morph not observed. Conidiomata pycnidial on PDA, globose to clavate, solitary or aggregated in clusters, semi-immersed, black; with dark brown conidial masses. Conidiophores reduced to conidiogenous cells. Conidiogenous cells hyaline, smooth, ampulliform to lageniform, 5–15 × 2–6 μm. Conidia straight to slightly curved, ellipsoid, 4-septate, 18.5–25.5 × 5–7.5 μm (mean = 22 × 6 μm, n = 30); basal cell conic, thin-walled, 3–7 μm long; three median cells doliiform, pale to dark brown, septa darker than the rest of the cell, 11–16 μm long (second cell from base pale brown, 3.5–6.5 μm long; third cell dark brown, 3.5–8 μm long; fourth cell brown, 3.5–7 μm long); apical cell hyaline, subcylindrical, 3–5 μm long; with 2–3 tubular apical appendages, unbranched, 15–33 μm long (mean = 23); basal appendage centric, single, unbranched, tubular, 4–7.5 μm long (mean = 5).

#### Culture characteristics.

Colonies on PDA reach 30–40 mm in diameter after 7 days of growth at 25 °C under 12 h daylight, cottony, with moderate aerial mycelium on the surface, edge undulate. The upper view is whitish with a black fruiting body. The reverse is yellow to pale honey-colored, with black, gregarious conidiomata.

#### Material examined.

Thailand • Chiang Rai Province, Mueang Chiang Rai District, Ban Du, the fruit of rambutan (*Nepheliumlappaceum*, June 2023, Maryam Fallahi, dried culture L2-7 (MFLU 24-0259), living culture MFLUCC 24-0254; Thailand, Chiang Rai Province, Mueang Chiang Rai District, Doi Hang, on lemon drop mangosteen (*Garciniaintermedia*), January 2023, Maryam Fallahi, dried culture MF11-3 (MFLU 24-0257), living culture MFLUCC 24-0233.

#### Notes.

**Figure 28.**
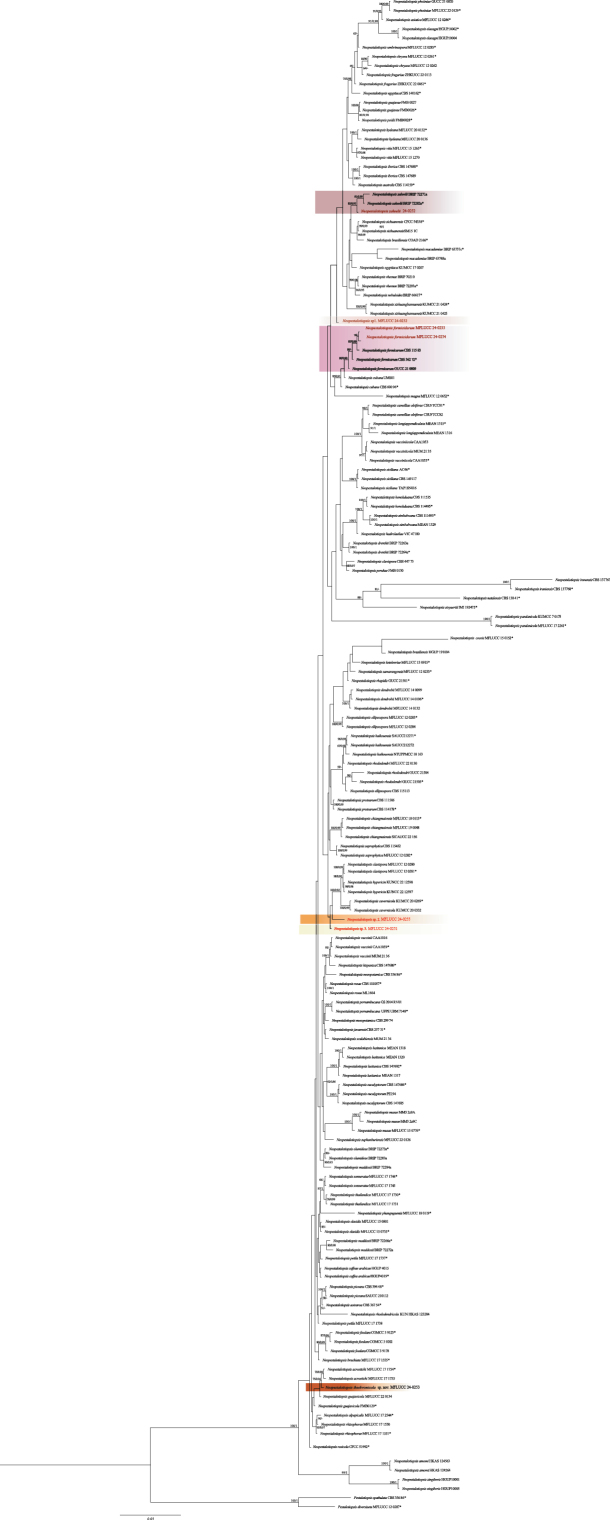
Phylogenetic tree of the *Neopestalotiopsis* ssp. generated by Bayes analysis of combined ITS, *tef1*, and *tub2* sequence data. The ultrafast maximum likelihood (ML) bootstrap support values ≥ 50% (BT) and Bayesian posterior probabilities ≥ 0.95 (BYPP) are shown, respectively, near the nodes. The ex-type strains are marked with an asterisk. The tree is rooted in *Pestalotiopsisspathulate* (CBS 356 86) and *Pestalotiopsisdiversiseta* (MFLUCC 12 0287).

The strain MFLUCC 24-0254 and MFLUCC 24-0233 clustered with strains of *Neopestalotiopsisformicidarum* in the same subclade with 80% ML bootstrap support and 0.90 BYPP (Fig. 28). The base pair differences between *N.formicidarum* strains MFLUCC 24-0254 and ex-type CBS:362.72 revealed a 0.79% nucleotide difference in ITS (4/506 bp) and a 0.75% difference in *tub2* (3/445 bp). They showed no difference in *tef1. Neopestalotiopsisformicidarum* (MFLUCC 24-0254) is similar to the ex-type strain of *N.formicidarum* (CBS:362.72) (Maharachchikumbura et al. 2014). However, it produces slightly shorter conidia than that of the ex-type strain with an L/W ratio = 3.6 (18.5–25.5 × 5–7.5 μm (mean = 22 × 6 μm) in *N.formicidarum* (MFLUCC 24-0254) vs. 21–28 × 7.5–9.5 μm (mean = 24.6 × 8.6 μm) in *N.formicidarum* (CBS 362.72, ex-type)). *Neopestalotiopsisformicidarum* was first reported as a saprobic species collected from dead ants in Ghana and plant debris from Cuba (Maharachchikumbura et al. 2014). This is the first host record on rambutan and lemon drop mangosteen for *N.formicidarum* worldwide.

**Figure 29.**
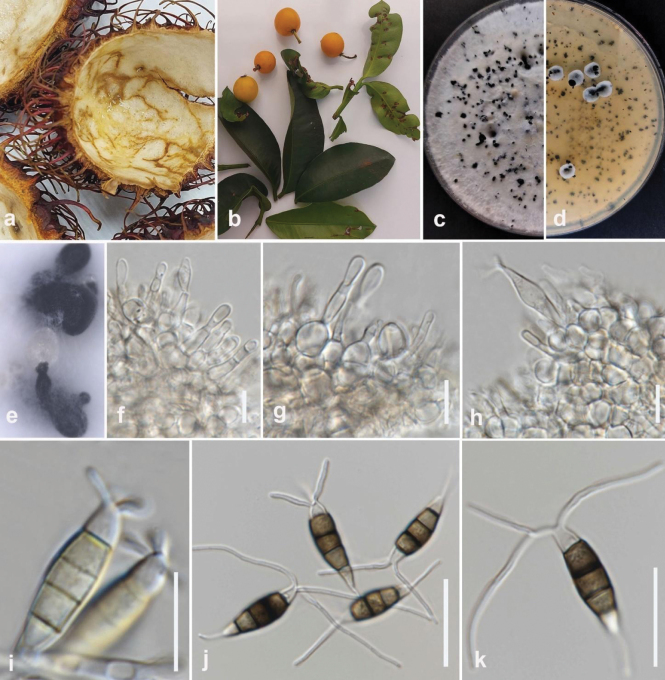
*Neopestalotiopsisformicidarum* (MFLUCC 24-0254) **a** fruit lesion on rambutan **b** dry leaf spot on lemon drop mangosteen (*Garciniaintermedia*) **c** front, and **d** back views of the colony on PDA after 60 days at 25 °C **e** conidiomata **f–h** conidiogenous cells **i–k** conidia. Scale bars: 10 μm (**f–h**); 20 μm (**I–k**).

### ﻿ Neopestalotiopsis zakeelii

Taxon classificationFungiSordariomycetesPestalotiopsidaceae

Prasannath, Akinsanmi & R.G. Shivas, Journal of Fungi 7: 12 (2021)

6917E7AC-CE37-5599-8125-29EA9EB04784

Index Fungorum: IF840920

Fig. 30

#### Description.

Pathogenic to persimmon (*Diospyrosehretioides*) and causes dark brown leaf spots. Sexual morph not observed. Conidiomata pycnidial on PDA, scattered, aggregated, immersed, or semi-immersed, with black conidial mass. Conidiophores reduced to conidiogenous cells. Conidiogenous cells hyaline, smooth, ampulliform to lageniform, 4–15 × 3–5 μm. Conidia medium to dark brown, fusiform to ellipsoidal, straight or curved, 4-septate, 20–30 × 5–8 μm (mean = 22 × 6 μm, n = 30); basal cell conical, 2–4 μm long (mean = 3 μm), hyaline, smooth, thin-walled; basal appendage filiform, unbranched, centric, 2–4 μm long; three median cells doliiform, 11–17.5 μm (mean = 16 μm), smooth, septa darker than the rest of the cell (second cell from basal cell olivaceous to brown, 3.5–7 μm long (mean = 5.5 μm); third cell brown to dark brown, 3.5–7 μm long (mean = 5.3 μm); fourth cell medium brown, 4–7 μm long (mean = 5.5 μm)); apical cell conical to subcylindrical, 2–5 μm long (mean = 3 μm), hyaline, smooth, thin-walled, with 2–3 tubular apical appendages (mostly 2), unbranched, filiform, 6–21.5 μm long (mean = 12 μm). Basal appendage single, unbranched, tubular, centric, 2–8 μm long.

**Figure 30.**
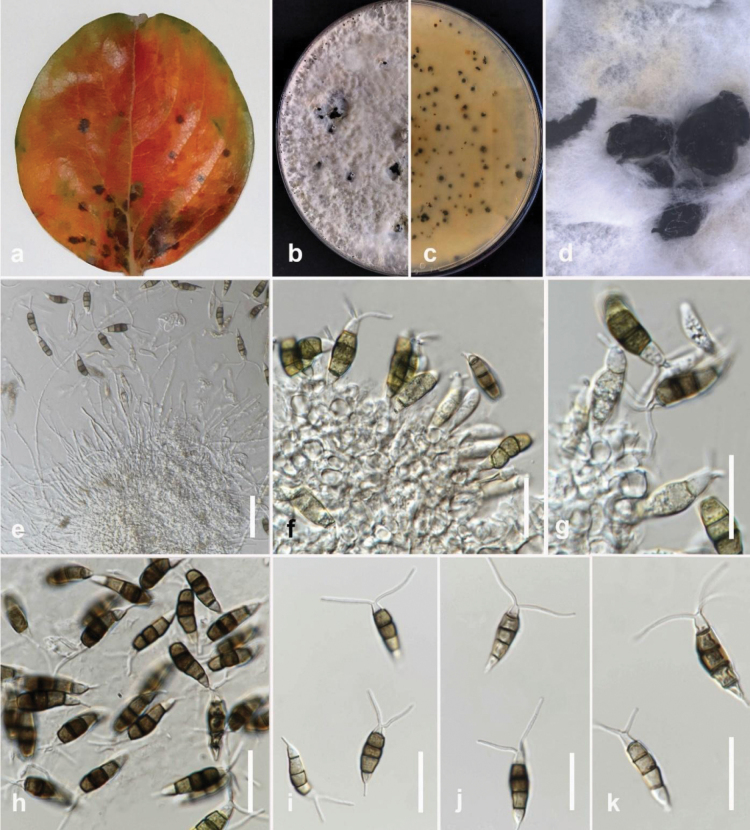
*Neopestalotiopsiszakeelii* (MFLUCC 24-0252) **a** leaf spot on persimmon **b** front, and **c** back views of the culture on PDA after 60 days at 25 °C **d** conidiomata **e–g** conidiophore, and conidiogenous cells **h–k** conidia. Scale bars: 20 μm.

#### Culture characteristics.

Colonies on PDA reach 55–65 mm in diameter after 7 days of growth at 25 °C under 12 h daylight, cottony, with abundant white aerial mycelium. Upper view white and the reverse primrose. Yellow pigment and black fruiting bodies appear with age on the agar medium.

#### Material examined.

Thailand • Chiang Rai Province, Mueang Chiang Rai District, Doi Hang, leaf spots on persimmon (*Diospyrosehretioides*), February 2023, Maryam Fallahi, dried culture MF54-1 (MFLU 24-0256), living culture MFLUCC 24-0252.

#### Notes.

Strain MFLUCC 24-0252 clustered in the same subclade with the strains of *Neopestalotiopsiszakeelii* (BRIP 72282a, holotype) with 85% ML bootstrap support and 0.95 BYPP (Fig. 28). The base pair differences between *N.zakeelii* strains MFLUCC 24-0252 and BRIP 72282a revealed a 0.46% (2/435 bp) nucleotide difference in *tef* and *tub2* and no differences in ITS. *Neopestalotiopsiszakeelii* (MFLUCC 24-0252) is similar to the holotype of *N.zakeelii* in morphology. It was first reported from flower blight of *Macadamiaintegrifolia* in Australia (Prasannath et al. 2021). This study provides a new host and geographical record for *N.zakeelii* on persimmon in Thailand.

### ﻿ Neopestalotiopsis theobromicola

Taxon classificationFungiSordariomycetesPestalotiopsidaceae

Fallahi, Jayawar. & K.D. Hyde sp. nov.

249134F3-5869-5EF5-AE6E-3EBDE1D68496

Index Fungorum: IF903267

Fig. 31

#### Etymology.

‘*theobromicola*’ refers to the host plant genus from which the fungus was isolated.

#### Description.

Associated with leaf spots of cacao (*Theobromacacao*). Sexual morph not observed. Conidiomata acervularon on PDA, aggregated and scattered, immersed and semi-immersed in agar medium, exuding black conidial mass. Conidiophores reduced to conidiogenous cells. Conidiogenous cells hyaline to pale brown, subcylindrical to ampuliform, 3–7 × 2–5 μm. Conidia clavate or fusiform, straight or slightly curved, yellow-brown to brown, 4 septate, 20–30 × 5–7.5 μm (mean = 26 × 6.5 μm, n = 30). Basal cell hyaline, conoid, with truncate base, 3.5–8 μm long; median cells, versicolored, darker than other cells, 14–19 μm long (mean = 17 μm, n = 30) (second cell from the base yellow-brown, 4–6.8 μm long; third cell from the base medium brown, 4.6–7 μm long; fourth cell from the base pale to medium brown, 4–7 μm long); apical cell hyaline, conic, 3–6 μm with 2–3 tubular apical appendages, hyaline, filiform, unbranched, and 15–29 μm long (mean = 22.5 μm, n = 30); basal appendage tubular, unbranched, solitary, hyaline, and 2–5.5 μm long.

**Figure 31.**
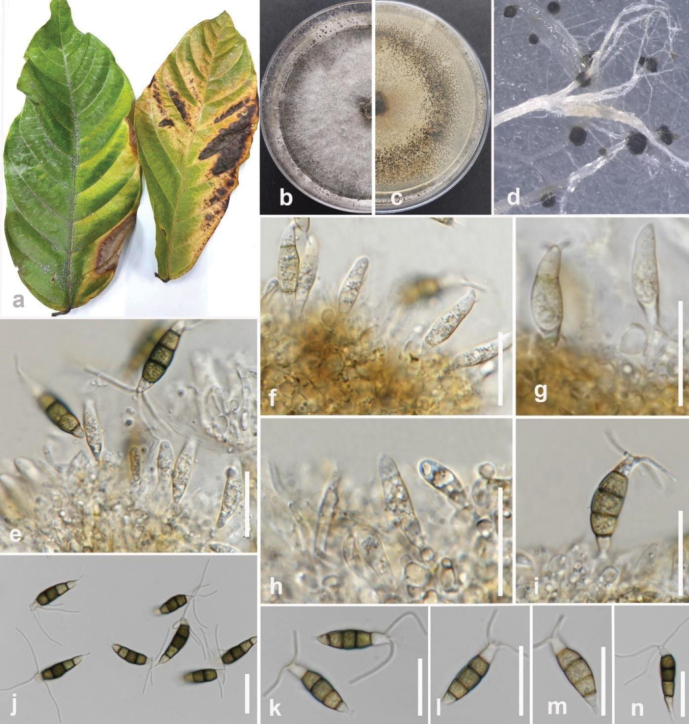
*Neopestalotiopsistheobromicola* (MFLUCC 24-0253, holotype) **a** leaf spots in cacao**b** front, and **c** back views of the colony on PDA after 30 days at 25 °C **d** conidiomata on SNA **e–i** conidiophore and conidiogenous cells **j–n** conidia. Scale bars: 20 μm.

#### Culture characteristics.

Colonies on PDA reach 65–70 mm in diameter after seven days of growth at 25 °C under 12 h daylight, white with moderate aerial mycelium, shape irregular, edge undulated, margin fluffy, with black conidial mass. The upper view is white, reverse honey-colored with age.

#### Material examined.

Thailand • Chiang Rai Province, Phan District, Sai Khao, on leaf spots in cacao (*Theobromacacao*), February 2023, Maryam Fallahi, dried culture MF115-1 (MFLU 24-0258, holotype), ex-holotype culture MFLUCC 24-0253.

#### Notes.

Strain MFLUCC 24-0253 formed an independent branch in the subclade, including the strains and ex-type strains of *Neopestalotiopsisacrostichi* and *N.guajavicola* with 75% ML bootstrap support and 0.94 BYPP (Fig. 28), and is introduced here as a new species, namely *Neopestalotiopsistheobromicola*. The base pair differences between *N.theobromicola* strains MFLUCC 24-0253 (holotype) and MFLUCC 17-1754 (ex-type) revealed a 0.40% nucleotide difference in ITS (2/504 bp, 3 gaps) and no difference in *tef1* and *tub2*. The base pair differences between *N.theobromicola* (ex-type strain MFLUCC 24-0253) and ex-type strains of *N.guajavicola* (FMBO 129) revealed a 0.20% nucleotide difference in ITS (1/506 bp), 0.26% differences in *tef1* (1/426 bp), and 0.25% differences in *tub2* (1/396 bp). *Neopestalotiopsistheobromicola* (ex-type strain MFLUCC 24-0253) differs from the type strain of *N.acrostichi* by having the conidia with 2-3 apical appendages (*N.acrostichi*: conidia with 3-5 apical tubular appendages, (16–)19–28. 5(–33. 5) μm long) (Norphanphoun et al. 2019). *Neopestalotiopsistheobromicola* (MFLUCC 24-0253) differs from the type strain of *N.guajavicola* by having larger conidia with an L/W ratio = 4 (26 × 6.5 μm in *N.theobromicola* (ex-type strain MFLUCC 24-0253) vs. 23.3 ± 1.6 × 6.5 ± 0.5 μm in *N.guajavicola* (FMBO 129)) (UI Haq et al. 2021).

### ﻿ Neopestalotiopsis

Taxon classificationFungiSordariomycetesPestalotiopsidaceae

sp. 1

19589A0D-2584-5EB7-BFF3-5705A2AF9CB4

Fig. 32

#### Description.

Pathogenic to sapodilla sapote (*Manilkarazapota*) and causes circular to irregular brown lesions on leaves. Sexual morph not observed. Conidiomata acervular on PDA, solitary or aggregated, semi-immersed in agar medium, containing a dark mass of conidia. Conidiophores reduced to conidiogenous cells. Conidiogenous cells ampulliform to lageniform, hyaline, 3–15 × 2–5 μm. Conidia fusiform or spindle-shaped, straight to slightly curved, 4-septate, 15–29 × 4–6.5 μm (mean = 22.4 × 5.3 μm, n = 30); basal cell conical, hyaline to pale brown, thin-walled, 3–5.6 μm long; the three middle cells brown to dark brown, with septa that are darker than the other cells, and 9–19 μm long (mean = 13.5 μm, n = 30). The second cell from the base 4–6.8 μm long; the third cell from the base 3.8–7.8 μm long; the fourth cell from the base 3.5–5.8 μm long; the apical cell conical, hyaline, thin-walled, 2.7–5 μm long, with 2–3 apical appendages (mostly 2), tubular, hyaline, unbranched, and 12.8–26 μm long. Basal appendage filiform, hyaline, unbranched, singular, and 3–9 μm long.

**Figure 32.**
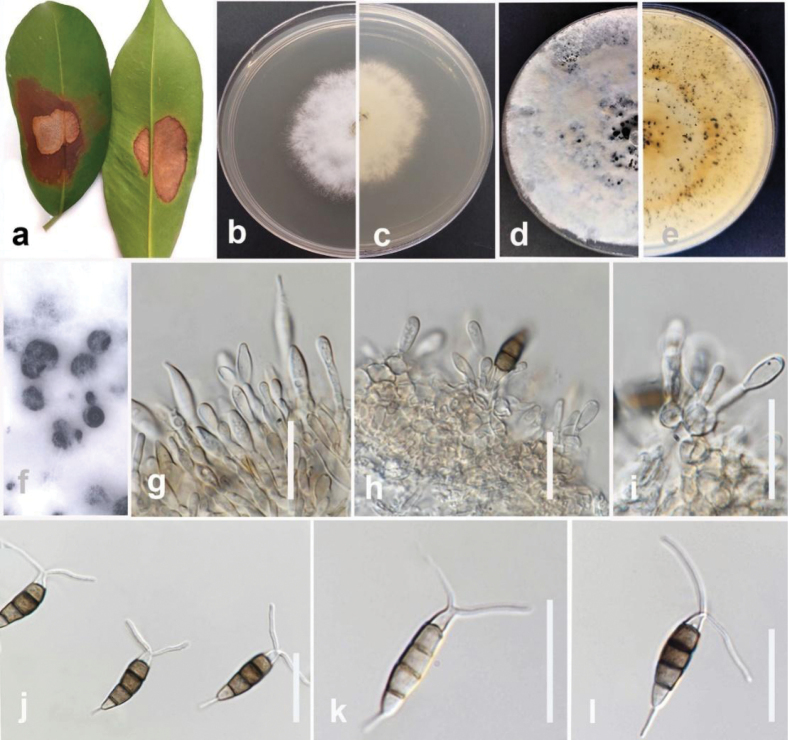
*Neopestalotiopsis* sp. 1 (MFLUCC 24-0232) **a** leaf spots in sapodilla sapote **b** front, and **c** back views of the colony on PDA after 7 days **d** front and **e** back view of the colony on PDA after 60 days at 25 °C **f** conidiomata **g–i** conidiophore and conidiogenous cells **j–l** conidia. Scale bars: 20 μm.

#### Culture characteristics.

Colonies on PDA reach 40–50 mm in diameter after seven days of growth at 25 °C under 12 h daylight, cottony, shape regular, circular. The upper view is white, and the reverse has yellow pigmentation, with black fruiting body clusters arising with age.

#### Material examined.

Thailand • Chiang Rai Province, Mueang Chiang Rai District, Doi Hang, leaf spots on sapodilla sapote (*Manilkarazapota*), December 2022, Maryam Fallahi, dried culture MF10-1 (MFLU 24-0254), living culture MFLUCC 24-0232.

#### Notes.

Based on phylogenetic analysis of ITS, *tub2*, and *tef1* sequence data, strain MFLUCC 24-0232 forms an independent branch with no bootstrap support (Fig. 28). Therefore, the MFLUCC 24-0232 was kept as an unidentified species, *Neopestalotiopsis* sp. 1 on sapodilla sapote, until further collections become available.

### ﻿ Neopestalotiopsis

Taxon classificationFungiSordariomycetesPestalotiopsidaceae

sp. 2

91BAA47F-8C71-5C19-BC2A-58DFACAC7106

Fig. 33

#### Description.

Pathogenic to mangosteen (*Garciniamangostana*), causes dark, sunken lesions on the fruit surface, often starting near wounds or natural openings. Sexual morph not observed. Conidiomata acervular on PDA, solitary or aggregated, immersed or semi-immersed in agar medium, containing dark mass of conidia. Conidiophores reduced to conidiogenous cells. Conidiogenous cells ampulliform to lageniform, and hyaline, 3–8 × 2–5 μm. Conidia fusiform, 4-septate, straight or slightly curved, 17–26 × 3.5–6.7 μm (mean = 21 × 5 μm, n = 40); basal cell conic, hyaline, 3–5.4 μm long; three median cells 12–18 μm long, brown to dark brown, septa darker than the rest of the cell; second cell from base brown, 3.5–6 μm long; third cell dark brown, 3–6 μm long; fourth cell darker, 2.7–6.5 μm long; apical cell 2.7–5.4 μm long, conic, hyaline, smooth-walled, with 2–3 (mostly 3) tubular apical appendages, 5.5–20 μm long. Basal appendage single, unbranched, tubular, centric, 3.9–8 μm long.

**Figure 33.**
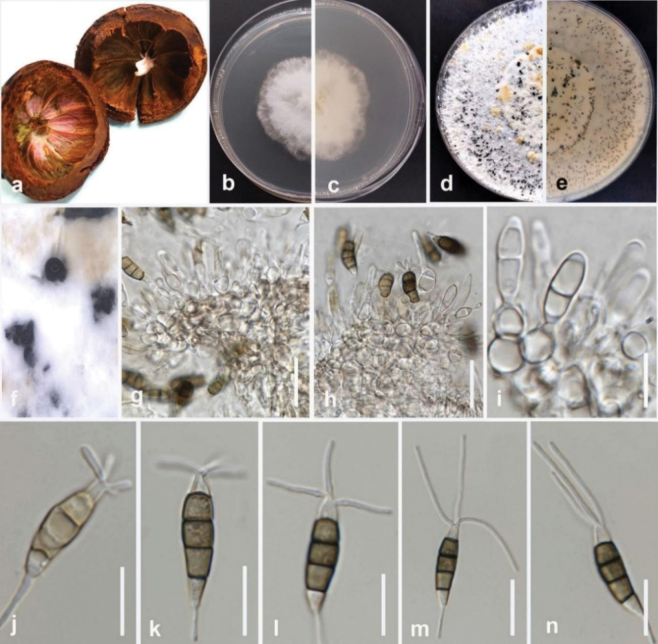
*Neopestalotiopsis* sp. 2 (MFLUCC 24-0255) **a** fruit rot on mangosteen **b** front, and **c** back views of the colony on PDA after 7 days **d** front and **e** back view of the colony on PDA after 60 days at 25 °C **f** conidiomata **g–i** conidiophore and conidiogenous cells **j–n** conidia. Scale bars: 10 μm.

#### Culture characteristics.

Colonies on PDA reach 40–50 mm in diameter after 7 days of growth at 25 °C under 12 h daylight, cottony, with an irregular shape, edge undulate, aerial mycelium on the surface. Upper view white and the reverse primrose. Yellow pigment and black fruiting bodies appear with age.

#### Material examined.

Thailand • Chiang Rai Province, Mueang Chiang Rai District, Ban Du, the fruit of mangosteen (*Garciniamangostana*), June 2023, Maryam Fallahi, dried culture L3-4 (MFLU 24-0260), living culture MFLUCC 24-0255.

#### Notes.

Phylogenetic analysis based on ITS, *tef1*, and *tub2* sequence data revealed that strain MFLUCC 24-0255 from the present study forms an independent subclade close to strains and type material of *Neopestalotiopsisclavispora*, *N.cavernicola*, and strain MFLUCC 24-0251 with no bootstrap support (Fig. 28); hence, we keep it as an unidentified species, *Neopestalotiopsis* sp. 2, until more collections become available. The base pair differences between *Neopestalotiopsis* sp. 2 (MFLUCC 24-0255), *N.clavispora* (MFUCC 12-0281), *N.cavernicola* (KUMCC 20-0269, ex-type), and *Neopestalotiopsis* sp. 3 (MFLUCC 24-0251) are presented in Table 5. Based on asexual morph and morphology (Table 7), *Neopestalotiopsis* sp. 2 (MFLUCC 24-0255) is different from the two well-known species mentioned above (Liu et al. 2021, Maharachchikumbura et al. 2012) as well as from *Neopestalotiopsis* sp. 3 (MFLUCC 24-0251).

**Table 5.** The base pair differences between *Neopestalotiopsis* sp. 2 (MFLUCC 24-0255) and *N.clavispora* (MFUCC 12-0281), *N.cavernicola* (KUMCC 20-0269, ex-type strain), and *Neopestalotiopsis* sp. 3 (MFLUCC 24-0251).

		*N.cavernicola* (KUMCC 20-0269, ex-type)	*N.clavispora* (MFLUCC12-0281, ex-type)	*Neopestalotiopsis* sp. 3 (MFLUCC 24-0251)
***Neopestalotiopsis* sp. 2 (MFLUCC 24-0255)**	ITS	0%	0%	0%
	*tef1*	1.9% (8/422 bp)	1.2% (5/422 bp)	0.95% (4/422 bp)
	*tub2*	0%	0.75% (3/401 bp)	0%

### ﻿ Neopestalotiopsis

Taxon classificationFungiSordariomycetesPestalotiopsidaceae

sp. 3

8CDC8505-74D4-597C-AA20-97DE4DBEBCEF

Fig. 34

#### Description.

Pathogenic to guava (*Psidiumguajava*), and causes small brown leaf spots. Sexual morph not observed. Conidiomata acervular on PDA, solitary or aggregated, immersed or semi-immersed in agar medium containing the dark mass of conidia. Conidiophores reduced to conidiogenous cells. Conidiogenous cells ampulliform to lageniform, hyaline, 3–8 × 2–4 μm. Conidia, olivaceous to yellow-brown, fusiform, straight or slightly curved, 4-septate, 21–29 × 4.5–6.5 μm (mean = 26 × 5.7 μm, n = 40); basal cell conic, hyaline, 4–7.3 μm long; three median cells 13–20.5 μm long, olivaceous to yellow-brown, septa darker than the rest of the cell; second cell from base yellow-brown, 4.5–9 μm long; third cell olivaceous, 4–7 μm long; fourth cell darker, 3–7 μm long; apical cell 4–5.5 μm long, conic, hyaline, smooth-walled, with 2–3 (mostly 2) tubular apical appendages, 3.5–18 μm long. Basal appendage single, unbranched, tubular, centric, 4–8 μm long.

**Figure 34.**
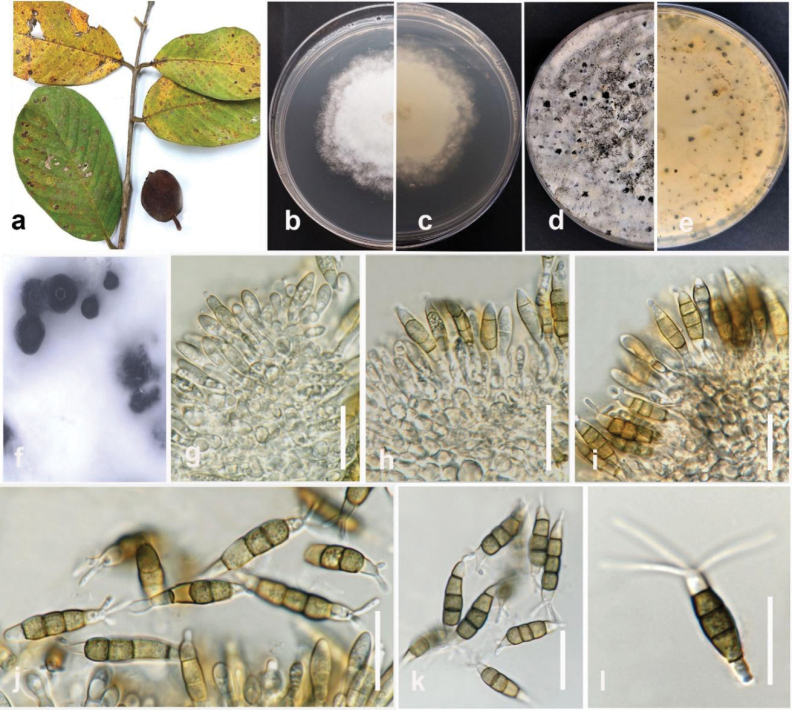
*Neopestalotiopsis* sp. 3 (MFLUCC 24-0251) **a** leaf spots in guava **b** front and **c** back views of the colony on PDA after 7 days **d** front and **e** back view of the colony on PDA after 60 days at 25 °C **f** conidiomata **g–i** conidiophore and conidiogenous cells **j–l** conidia. Scale bars: 20 μm

#### Culture characteristics.

Colonies on PDA reach 60–70 mm in diameter after 7 days of growth at 25 °C under 12 h daylight, white, edge undulate, with aerial mycelium on the surface, and black fruiting bodies. The reversing turn pale luteous with age.

#### Material examined.

Thailand • Chiang Rai Province, Phan District, Sai Khao, leaf spots on guava (*Psidiumguajava*), February 2023, Maryam Fallahi, dried culture MF107-1 (MFLU 24-0255), living culture MFLUCC 24-0251.

#### Notes.

Based on phylogenetic analysis of ITS, *tub2*, and *tef1* sequence data, strain MFLUCC 24-0251 formed an independent subclade close to strains and type material of *Neopestalotiopsisclavispora*, *N.cavernicola*, and *Neopestalotiopsis* sp. 2 (MFLUCC 24-0255) with no bootstrap support (Fig. 28), hence it was kept as an unidentified species, *Neopestalotiopsis* sp. 3. The base pair differences between *Neopestalotiopsis* sp. 3 (MFLUCC 24-0251) and *N.clavispora* (MFUCC 12-0281), *N.cavernicola* (KUMCC 20-0269, ex-type strain), and *Neopestalotiopsis* sp. 2 are presented in Table 6. Based on conidial morphology and colour, the length of conidia, and apical and basal appendage (Maharachchikumbura et al. 2012; Liu et al. 2021), *Neopestalotiopsis* sp. 3 (MFLUCC 24-0251) differs from the two above-mentioned species (Table 7).

**Table 6.** The base pair differences between *Neopestalotiopsis* sp. 3 (MFLUCC 24-0251 and *N.clavispora* (MFUCC 12-0281), *N.cavernicola* (KUMCC 20-0269, ex-type strain), and *Neopestalotiopsis* sp. 2 (MFLUCC 24-0255).

		*N.cavernicola* (KUMCC 20-0269, ex-type)	*N.clavispora* (MFLUCC12-0281, ex-type)	*Neopestalotiopsis* sp. 2 (MFLUCC 24-0255
***Neopestalotiopsis* sp. 3 (MFLUCC 24-0251)**	ITS	0%	0%	0%
	*tef1*	0.95% (4/422 bp)	0.24% (1/422 bp)	0.95% (4/422 bp)
	*tub2*	0%	0.75% (3/422 bp)	0%

**Table 7.** Characteristics of *Neopestalotiopsis* sp. 2 (MFLUCC 24-0255), *Neopestalotiopsis* sp. 3 (MFLUCC 24-0251) from the present study, and the type strains of *N.clavispora* (MFLU12-0418, epitype) and *N.cavernicola* (KUMCC 20-0269, ex-type).

	Conidiophores and conidiogenous cells	Conidial colour	Conidial length	Apical appendage	Basal appendage	Colony	Growth rate (7 days)
***Neopestalotiopsis* sp. 2**	Conidiophores reduced to conidiogenous cells, conidiogenous cells ampulliform to lageniform	brown to dark brown	21 × 5 μm	tubular (mostly 3), 5.5–20 μm	Tubular, 3.9–8 μm long	white, edge undulate, reverse primrose	40–50 mm
***Neopestalotiopsis* sp. 3**	Conidiophores reduced to conidiogenous cells, conidiogenous cells ampulliform	olivaceous to yellow-brown	28 × 6 μm	tubular (mostly 2), 5–18 μm	Tubular,3–6 μm long	white, edge undulate, reverse pale luteous	60–70 mm
***Neopestalotiopsisclavispora***	Conidiogenous cells, hyaline, simple, short or relatively long, filiform	dark brown to olivaceous	22 × 7.2 μm	tubular (mostly 3), 22–33 μm	Filiform, 3–5.5 μm long	whitish, edge undulate, reverse pale luteous	70 mm
***Neopestalotiopsiscavernicola***	Conidiophores up to 25 μm, discrete, cylindrical, hyaline to pale brown, smooth	yellow-brown to brown	22.41 × 7.89 μm	tubular (2–4), 5–30 μm	filiform, tubular, 2.5–9 μm long	white, edge undulate, reverse pale yellow	no data

##### ﻿Pathogenicity assay

Pathogenicity tests were conducted to verify the disease-causing capabilities of the strains of *Colletotrichum* (five strains), *Diaporthe* (six strains), *Fusarium* (five strains), and *Neopestalotiopsis* (five strains).

**Figure 35.**
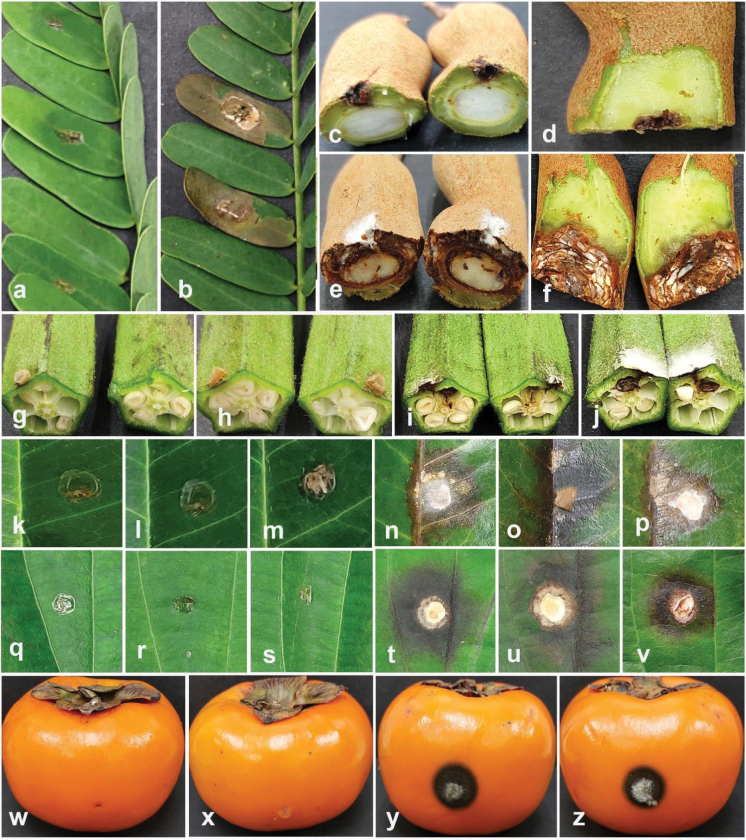
Pathogenicity tests of five strains of *Colletotrichum* spp. on various hosts **a–e** pathogenicity tests of *C.makassarense* (MFLUCC 24-0237) on leaves and fruits of tamarind **a** controls (leaves) were assayed after three days **b** inoculated leaves after three days **c** controls (fruits) were assayed after two weeks **d, e** inoculated fruits after two weeks **f–i** pathogenicity tests of *C.abelmoschi* (MFLUCC 24-0239) on okra **f, g** controls were assayed after two weeks **h, i** inoculated fruits after two weeks **j–o** pathogenicity tests of *C.fructicola* (MFLUCC 24-0240) on leaves of jackfruit **j–l** controls were assayed after one week **m–o** inoculated leaves after one week **p–u** pathogenicity tests of *C.siamense* (MFLUCC 24-0235) on leaves of black pepper **p–r** controls were assayed after one week **s–u** inoculated leaves after one week **v–y** pathogenicity tests of *C.plurivorum* (MFLUCC 24-0238) on fruits of persimmon **v, w** controls were assayed after five days **x, y** inoculated fruits after five days.

Pathogenicity tests were performed on five strains of *Colletotrichum* spp., and all strains demonstrated pathogenicity towards their respective hosts, whereas the controls exhibited no symptoms (Fig. 35). Koch’s postulates were validated through the re-isolation of the same fungi and confirmation of their colony and morphological characteristics.

**Figure 36.**
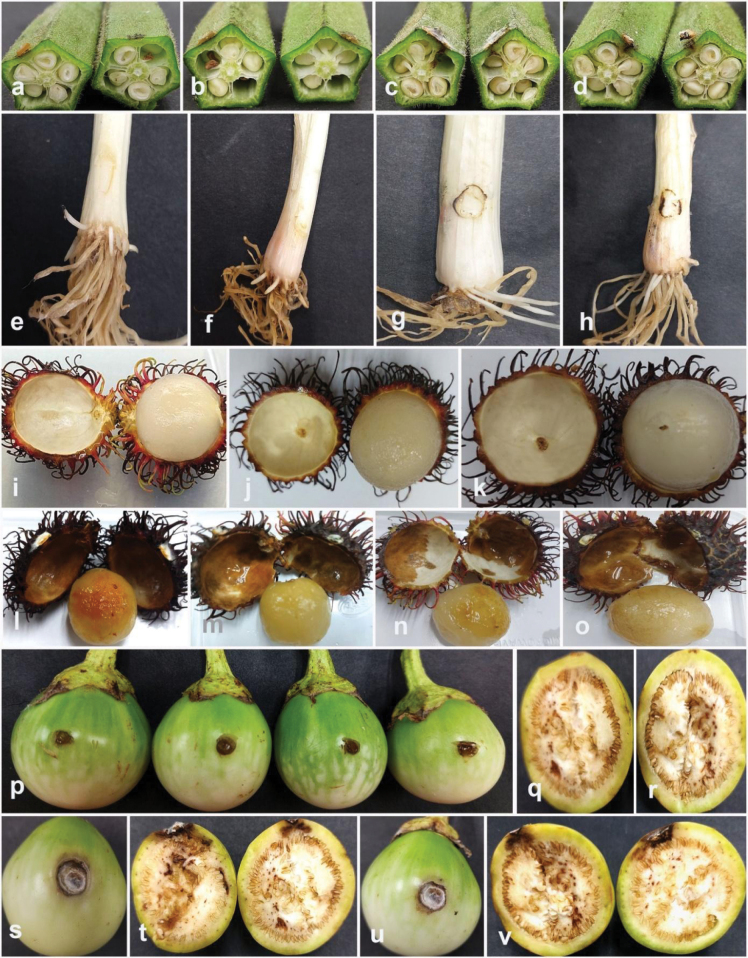
Pathogenicity tests of six strains of *Diaporthe* spp. on various hosts **a–d** pathogenicity tests of *D.sennicola* (MFLUCC 24-0241) on okra **a, b** controls were assayed after two weeks **c, d** inoculated fruits after two weeks **e–h** pathogenicity tests of *D.fistulosi* (MFLUCC 24-0244) on spring onion **e, f** controls were assayed after one week **g, h** inoculated spring onions after one week **i–o** pathogenicity tests of *D.hongkongensis* (MFLUCC 24-0246) and *D.siamensis* (MFLUCC 24-0245) on rambutan **i–k** controls were assayed after one week **l–o** inoculated fruits by strain MFLUCC 24-0246 after one week. **n, o** inoculated fruits by strain MFLUCC 24-0245 after one week **p–v** pathogenicity tests of *D.rosae* (MFLUCC 24-0243) and *D.melongenicola* (MFLUCC 24-0242) on fruits of makhuea **p–r** controls were assayed after two weeks **s, t** inoculated fruits by strain MFLUCC 24-0243 after two weeks **u, v** inoculated fruits by strain MFLUCC 24-0242 after two weeks.

Pathogenicity tests were carried out on six strains of *Diaporthe* spp., and all these strains demonstrated varying degrees of pathogenicity towards their host, while the controls remained symptom-free (Fig. 36). Koch’s postulates were validated through the re-isolation of the same fungi and confirmation of their colony and morphological characteristics.

**Figure 37.**
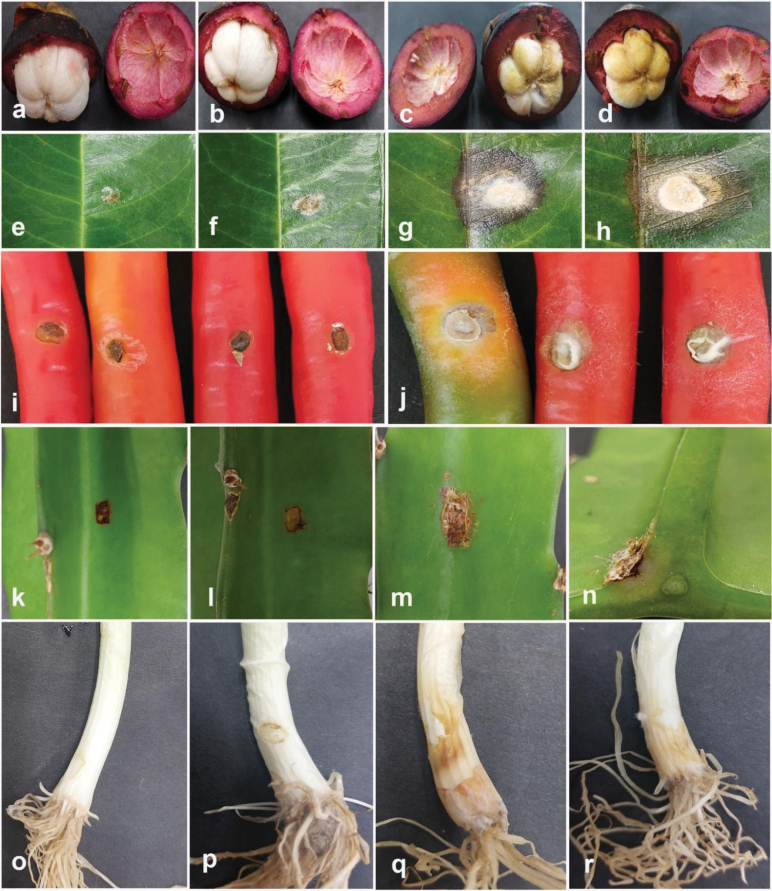
Pathogenicity tests of five strains of *Fusarium* spp. on various hosts. **a–d** Pathogenicity tests of *F.sulawesiense* (MFLUCC 24-0250) on mangosteen **a, b** controls were assayed after two weeks **c, d** inoculated fruits after two weeks **e–h** pathogenicity tests of *F.tanahbumbuense* (MFLUCC 24-0231) on leaves of durian **e, f** controls were assayed after five days **g, h** inoculated leaves after five days **i, j** pathogenicity tests of *F.tanahbumbuense* (MFLUCC 24-0231) on fruits of pepper **i** controls were assayed after one week **j** inoculated peppers after one week **k–n** pathogenicity tests of *F.bubalinum* (MFLUCC 24-0230) on the stem of dragon fruit **k, l** controls were assayed after two weeks **m, n** inoculated stems after two weeks **o–r** pathogenicity tests of *F.nirenbergiae* (MFLUCC 24-0248) on spring onion stem **o, p** controls were assayed after one week **q, r** inoculated spring onion after one week.

Pathogenicity tests were carried out on five strains of *Fusarium* isolated from specific hosts, and all the strains were able to infect their hosts, while the controls displayed no symptoms (Fig. 37). Verification of Koch’s postulates was achieved by re-isolating the same fungi and confirming their colony and morphological characteristics.

**Figure 38.**
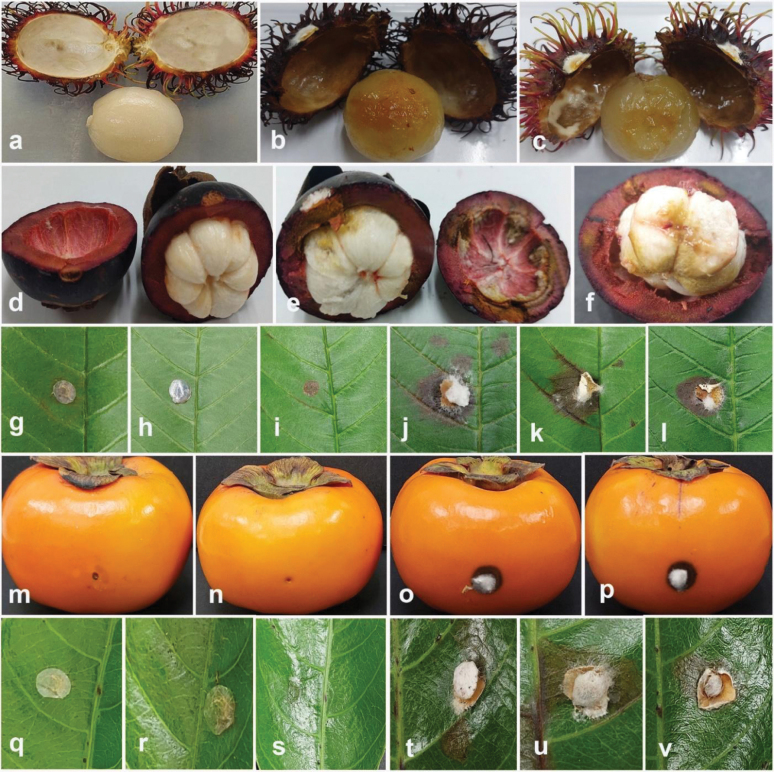
Pathogenicity tests of five strains of *Neopestalotiopsis* spp. on various hosts **a–c** pathogenicity tests of *N.formicidarum* (MFLUCC 24-0254) on rambutan **a** controls were assayed after one week **b, c** inoculated fruits after one week **d–f** pathogenicity tests of *Neopestalotiopsis* sp. 2 (MFLUCC 24-0255) on mangosteen **d** controls were assayed after two weeks **e, f** inoculated fruits after two weeks **g–l** pathogenicity tests of *Neopestalotiopsis* sp. 3 (MFLUCC 24-0251) on leaves of guava **g–I** controls were assayed after five days **j–l** inoculated leaves after five days **m–p** pathogenicity tests of *N.*zakeelii (MFLUCC 24-0252) on persimmon **m, n** controls were assayed after five days **o, p** inoculated fruits after five days **q–v** pathogenicity tests of *Neopestalotiopsis* sp. 1 (MFLUCC 24-0232) on leaves of sapodilla sapote **q–s** controls were assayed after five days **t–v** inoculated leaves after five days.

Pathogenicity tests were conducted on five strains of *Neopestalotiopsis* spp. isolated from various host plants, and all the strains demonstrated pathogenic effects on their hosts, while the controls did not exhibit any symptoms (Fig. 38). Koch’s postulates were substantiated through the re-isolation of the same fungi and confirmation of their colony and morphological characteristics.

## ﻿Discussion

This study provided critical insight into the diversity and impact of pathogenic fungi affecting several crops in northern Thailand. The identification of multiple fungal genera reveals the prevalence of plant-pathogenic fungi in tropical agricultural systems (Udayanga et al. 2012; Monkai et al. 2023). It highlights the ecological range of fungal species, such as *Fusariumsulawesiense* and *Neopestalotiopsisformicidarum*, which infect multiple plant hosts, complicating disease management and potentially causing significant economic losses (Marin-Felix et al. 2017; Jayawardena et al. 2019).

Several pathogenic strains of *Colletotrichum*, including *C.makassarense*, *C.abelmoschi*, *C.siamense*, *C.fructicola*, *C.plurivorum*, and *C.spaethianum*, have been isolated and identified from anthracnose and leaf spots. Pathogenicity tests confirmed their ability to induce disease symptoms, with *C.siamense* displaying an endophytic-to-pathogenic lifestyle shift, consistent with previous studies (Photita et al. 2001; Jayawardena et al. 2021). Furthermore, *Colletotrichumabelmoschi*, isolated from okra, was introduced as a novel species within the *C.gloeosporioides* species complex.

The study also identified pathogenic *Diaporthe* species associated with fruit spots, stem lesions, and wilting symptoms, including *D.hongkongensis*, *D.rosae*, *D.siamensis*, *D.sennicola*, *D.melongenicola*, and *D.fistulosi*. Pathogenicity tests confirmed their virulence, with *D.rosae*, which was previously identified as a saprobic species, now established as a pathogenic species on makhuea kheun (Wanasinghe et al. 2018). Historically, species classification was based on host specificity, leading to a proliferation of species names. However, recent phylogenetic analyses have shown that *Diaporthe* species are not host-specific (Dissanayake et al. 2017). While *Diaporthe* species are significant pathogens causing dieback in forest plants, research on this genus in Thailand remains limited, with 26 species reported from Thailand without molecular data (Cheewangkoon et al. 2021). This study updated the *Diaporthe* species causing diseases on tropical crops in Thailand using a polyphasic approach for precise identification.

Similarly, *Fusarium* species were isolated from diverse plant tissues as pathogens, saprotrophs, or endophytes (Gerlach and Nirenberg 1982). Notably, *F.tanahbumbuense* and *F.sulawesiense*, members of the *F.incarnatum* species complex, were first identified on *Musa* species in Indonesia (Maryani et al. 2019a). A concerning aspect is the ability of certain fungi, such as *Fusariumnirenbergiae*, to produce mycotoxins, raising food safety and public health concerns. Mycotoxigenic fungi are known contaminants of food products. Future research should focus on characterizing the mycotoxin profiles of *Fusarium* species identified in this study to assess their implications for food security and public health.

Additionally, the study documented several *Neopestalotiopsis* species primarily associated with leaf spots and fruit rots. *Neopestalotiopsisformicidarum*, *N.theobromicola*, *N.zakeelii*, and other unidentified species exhibited pathogenicity on rambutan and mangosteen fruits, confirming their role as significant plant pathogens in Thailand. These findings align with previous reports, confirming that *N.formicidarum* can act as an endophyte, saprobe, or pathogen (Maharachchikumbura et al. 2014; Tibpromma et al. 2018; UI Haq et al. 2021).

The species described in this study were identified from a diverse range of hosts, including tropical woody and herbaceous crops. The newly introduced species were recognized as pathogens on specific hosts but may possess broad host ranges, extensive geographic distribution, and the potential to transition between life modes. Consequently, future studies incorporating additional isolates and more precisely defined taxa are necessary to reassess and refine their host diversity and geographic distribution.

Overall, this study contributes to a better understanding of fungal pathogen distribution in northern Thailand by documenting new host-pathogen interactions and expanding their host ranges. It aligns with the recent research suggesting that plant-pathogenic fungi are evolving and adapting to new hosts, influenced by environmental changes and shifting agricultural practices. A multidisciplinary approach incorporating plant pathology, genetics, and climate science seems necessary to address the risks posed by these pathogens (Lombard et al. 2019; Aiello et al. 2021). Future efforts should focus on comprehensive monitoring strategies and integrated disease management approaches to minimize the impact of fungal pathogens and ensure agricultural sustainability (Photita et al. 2001; Jayawardena et al. 2021).

## Supplementary Material

XML Treatment for Colletotrichum

XML Treatment for Colletotrichum abelmoschi

XML Treatment for Colletotrichum fructicola

XML Treatment for Colletotrichum makassarense

XML Treatment for Colletotrichum siamense

XML Treatment for Colletotrichum plurivorum

XML Treatment for Colletotrichum spaethianum

XML Treatment for Diaporthe

XML Treatment for Diaporthe sennicola

XML Treatment for Diaporthe fistulosi

XML Treatment for Diaporthe hongkongensis

XML Treatment for Diaporthe rosae

XML Treatment for Diaporthe siamensis

XML Treatment for Diaporthe melongenicola

XML Treatment for Fusarium

XML Treatment for Fusarium bubalinum

XML Treatment for Fusarium sulawesiense

XML Treatment for Fusarium tanahbumbuense

XML Treatment for Fusarium languescens

XML Treatment for Fusarium nirenbergiae

XML Treatment for Neopestalotiopsis

XML Treatment for Neopestalotiopsis formicidarum

XML Treatment for Neopestalotiopsis zakeelii

XML Treatment for Neopestalotiopsis theobromicola

XML Treatment for Neopestalotiopsis

XML Treatment for Neopestalotiopsis

XML Treatment for Neopestalotiopsis

## ﻿Additional information

### Author ORCIDs

Maryam Fallahi https://orcid.org/0000-0002-6262-8771

Alireza Armand https://orcid.org/0000-0003-2455-3796

Fatimah Al-Otibi https://orcid.org/0000-0003-3629-5755

Kevin D. Hyde https://orcid.org/0000-0002-2191-0762

Ruvishika S. Jayawardena https://orcid.org/0000-0001-7702-4885

### Data availability

All of the data that support the findings of this study are available in the main text or Supplementary Information.

## Supplementary materials

Supplementary material 1 Supplementary data Data type xlsx https://binary.pensoft.net/file/1325310This dataset is made available under the Open Database License (http://opendatacommons.org/licenses/odbl/1.0/). The Open Database License (ODbL) is a license agreement intended to allow users to freely share, modify, and use this Dataset while maintaining this same freedom for others, provided that the original source and author(s) are credited.Maryam Fallahi, Alireza Armand, Fatimah Al-Otibi, Kevin D. Hyde, Ruvishika S. Jayawardena
